# The impact of official development assistance for health on health outcomes: a rapid systematic review

**DOI:** 10.1093/heapol/czaf102

**Published:** 2025-12-03

**Authors:** Newton Chagoma, Rohan Sweeney, Sumit Mazumdar, Marc Suhrcke

**Affiliations:** Centre for Health Economics, University of York, York, YO10 5DD England, United Kingdom; Department of Health Sciences, University of York, York, YO10 5DD England, United Kingdom; Centre for Health Economics, Monash Business School, Monash University, 900 Dandenong Road, Caulfield Campus, Victoria 3145, Australia; Centre for Health Economics, University of York, York, YO10 5DD England, United Kingdom; Centre for Health Economics, University of York, York, YO10 5DD England, United Kingdom; Luxembourg Institute of Socio-Economic Research, 11 Porte des Sciences, Maison des Sciences Humaines, L-4366 Esch-sur-Alzette/Belval, Luxembourg

**Keywords:** aid, development assistance for health, donors, aid effectiveness, health outcomes

## Abstract

Low- and middle-income countries (LMICs) have received substantial amounts of Official Development Assistance for Health (DAH) to address domestic health funding gaps and improve access to universal healthcare. However, the effectiveness of DAH in improving health outcomes remains contested, with varying findings across studies due to differences in methodologies, data sources, and target populations. This systematic review synthesizes the existing evidence on the impact of DAH on health outcomes in LMICs, highlighting both the positive and negative effects, and identifying key mechanisms through which aid influences health. A total of 61 studies were included in the review, with a primary focus on maternal and child health outcomes. Despite methodological differences, the weight of evidence indicates a generally positive impact of DAH, particularly in countries with higher governance standards and better economic conditions. Our findings underscore the importance of contextual factors, such as governance and proximity to aid-funded projects, in shaping the effectiveness of health aid. To maximize the impact of DAH, policymakers need to strengthen donor coordination, align aid with national health priorities, and reinforce domestic health systems. Future research should focus on refining causal inference methods and exploring innovative aid-delivery mechanisms to sustain long-term health improvements.

Key messages

- Development assistance for health (DAH) to low- and middle-income countries often faces criticism for being donor-driven, misaligned with local priorities, and administratively burdensome. These dynamics raise concerns about weak accountability, limited impact, and potential harm to local health systems, thereby fuelling ongoing debates about its effectiveness.
- While that may be true, extant evidence shows that DAH contributes to modest reductions in mortality and morbidity, and gains in life expectancy. However, the effect sizes are generally small and vary across context, outcome, and method.
- DAH tends to achieve stronger and more sustainable results when delivered through government systems and aligned with aid effectiveness principles like those in the Paris Declaration. Its impact is also highly heterogeneous, with greater effects in countries with stronger governance and health infrastructure.
- Future research must address persistent data and methodological gaps, including measurement errors and selective reporting, to strengthen causal claims and guide more effective DAH programming.

## Introduction

Official development assistance (ODA) remains highly relevant in low- and middle-income countries (LMICs), providing crucial support for socio-economic development and poverty reduction. It accounts for substantial revenues, not least in the health sector, where development assistance for health (DAH) disbursements have recently reached nearly $30 billion per year (IHME 2020). In low-income countries alone, DAH accounts for an average of around 30% of total health spending (WHO 2021). Advocates of health aid argue that it is vital in helping LMICs provide basic health care for all, in line with Sustainable Development Goal 3.8, and that it ultimately enhances population health (United Nations 2015, IHME 2020).

Yet aid, in general and health aid specifically, has also come under criticism. Among the earlier critics, Jepma (1995) noted that, while aid in general increased public consumption, it did not affect macroeconomic policies or growth in recipient countries. Similarly, Boone (1996) found no beneficial effect of aid for the poor but noted some positive influence on health in countries with better democracy and government transparency. Corruption, inefficiencies, bureaucratic failures and poor institutional development in LMICs have often been cited as grounds for the possibly limited productivity of aid (World Bank 1998, OECD 2005). More recently, calls to ‘decolonize’ aid have argued that aid channelled through Western systems, underpinned by Western perspectives, has even weakened recipient public sector—and indeed public health—systems (Kwete et al. 2022). To more comprehensively appreciate the existing evidence base, as well as its nuances, on the impact of DAH on health outcomes, this paper systematically evaluates the extant literature. The insights gained may in turn inform health financing policies and DAH programming in LMICs.

Although the ultimate goal of DAH is arguably to improve recipients’ health outcomes, its allocation is often determined by donors’ political and strategic preferences (Alesina and Dollar 2000). As a result, DAH may not necessarily be aligned with the disease burden and health needs in recipient countries (World Bank 1998, Hill 2002, Esser and Bench 2011, Paul and Vandeninden 2012, Dieleman et al. 2013, Van de Sijpe 2013). The proliferation of health aid from increasing numbers of donors has also introduced coordination challenges for recipient governments, including substantial time commitments to managing donors (Buse and Walt 1996, 1997). These limitations raised concerns about the effectiveness of health aid (Buse and Walt 1997, Hill 2002). The Paris Declaration on Aid Effectiveness (OECD 2005) provided a strategic ‘roadmap designed to improve the quality and developmental impact of aid’ based on the ‘Paris Principles’ for effective aid delivery, namely harmonization (of donor activities and procedures), alignment (with recipient priorities and management systems), and (recipient) country ownership of aid programmes (OECD 2005). Perhaps, at least in part, in response to these international developments, donor country electorates have increasingly demanded greater transparency and accountability regarding the impact of aid (Weiss et al. 2022). In addition, the seemingly mixed evidence base on the health impact of aid further stimulates the aid effectiveness debate.

Some studies have found significant positive aid effects in terms of reducing mortality rates (Chauvet et al. 2013, Bendavid 2014, Yogo and Mallaye 2015) and increasing life expectancy (Ziesemer 2016, Gutema and Mariam 2018), whereas others have not detected any impact (Kizhakethalackal 2009) or even found harmful effects on child mortality (Nwude et al. 2020). Some of these studies are quantitative, while others are based on qualitative assessments (Calleja and Cichocka 2022) or on reviews and expert opinions (Glennie 2020). These diverse methodologies, varied study settings, and aid-delivery channels can offer different perspectives and insights to researchers and policymakers. However, the heterogeneity across study findings makes it challenging to draw general conclusions regarding the effectiveness of health aid.

It is important to note that not all study designs can control for key sources of bias and confounding (Burguet and Soto 2013), particularly in the context of aid. Randomized controlled trials (RCTs) may also be less viable, as larger-scale aid is harder to randomize. As a result, it is difficult to determine the contexts in which DAH yields positive effects, and the debate about aid effectiveness continues (Gomanee et al. 2005, Pettersson 2007, Wolf 2007, Williamson 2008, Tarverdi and Rammohan 2017, Weiss et al. 2022).

This study employs a rapid systematic review methodology (Grant and Booth 2009). We seek to answer the question ‘What is the impact of health aid on health outcomes?’ by identifying and synthesizing quantitative studies that employed methods involving at least some credible effort at estimating a causal relationship between DAH and health. We critically examine the available evidence, highlighting what is known about DAH, when and where aid is effective, and why, and discuss the implications for global health policy, practice, and future research.

One previous systematic review by Taylor et al. (2013) focused on aid for reproductive, maternal, neonatal, and child health (RMNCH) programmes. It concluded that DAH had a modest positive effect on RMNCH outcomes. The authors also explored whether aid delivered in accordance with the Paris Principles was more effective but found insufficient evidence to make a definitive assessment at the time. A more recent and expansive review by the Centre for Healthy Development (CHD 2025) assessed over 2000 articles and found that, despite decades of substantial investment, the overall evidence on the effectiveness of DAH at global or national levels remains limited. The search strategy employed by CHD (2025) differs from ours by purposefully targeting impact evaluations of health aid delivered via a subset of aid channels to a subset of aid-recipient countries, whilst being less restrictive on study design, identifying predominantly ‘grey literature’ project evaluations. Consequently, the present reviews builds on these reviews, providing an updated and complimentary synthesis of the expanding evidence base, identifying a larger number of rigorous, peer-reviewed quantitative studies encompassing a broader range of health outcomes—from disease-specific indicators to all-cause mortality, morbidity and life expectancy. Further, we apply structured causal inference criteria and risk-of-bias assessments, allowing the generation of synthesis conclusions that give greater weight to studies with stronger methodological foundations. We also explore whether and how alignment with aid effectiveness principles influences health outcomes in recipient settings.

## Methods

### Eligibility criteria

We adapted the Morgan et al. (2018) PECO-S framework (Population Exposure Comparator Outcomes—Setting) to define the scope of this review and guide the search strategy. In contrast to Morgan et al. (2018), we replaced the descriptor ‘setting’ with ‘study design’, as this was deemed more suitable for our body of literature. Specifically, we chose the PECO-Study design because it provides a structured, transparent approach to defining the key elements of our review: population, exposure, comparator, outcomes, and study design/method. This approach is especially useful for studies that extend beyond clinical interventions and include broader public health and health systems research, where the choice of study design and methods can significantly influence the conclusions drawn.

#### Inclusion and exclusion criteria

We examined studies covering populations in LMICs to assess changes in health outcomes resulting from exposure to the level or types of DAH—financial and in-kind contributions in the form of grants, loans, and concessionary agreements provided to LMICs to support health development (Dieleman et al. 2015). This includes DAH that donors tie to specific purposes or give as general (untied) health-sector support, through on-budget (i.e. via the recipient government) or off-budget financing, from all sources and channels. The precise comparator is dependent on the study design, but it is generally a contemporaneous counterfactual. We also included studies looking at the health impact of overall ODA (which DAH is part of). For simplicity, we refer to the impact of DAH on health outcomes, including life expectancy, all-cause mortality rates, and disease-specific outcomes [such as mortality, disease prevalence and incidence, and disability-adjusted life years (DALYs)] throughout the remainder of this paper (see section Data synthesis and analysis, for more details).

Targeted studies included those that applied statistical/econometric methods to estimate the impact of DAH on health outcomes, thereby providing some degree of empirical causality and facilitating objective comparisons of effects across settings. Included study designs accounted for unobservable characteristics—panel data analysis methods, instrumental variables (IVs) analysis, generalized methods of moments (GMM), synthetic controls methods (SCM), regression discontinuity, difference-in-difference (DID), structural models, and control functions—or observable characteristics –propensity score and other matching methods, inverse probability doubly robust methods, regression adjustments and parametric regression on a matched sample. Studies were excluded if they were descriptive, qualitative, if full texts could not be retrieved, or if the publication was not in English. While qualitative research offers rich insights into the ‘how and why’ behind aid effectiveness, they were excluded in this review for two main reasons: first, our research question is specifically about the impact of DAH on health outcomes, implying a focus on causal relationships, i.e. does aid cause measurable changes in health outcomes. Thus, including qualitative studies would not directly answer our questions about the magnitude of aid’s impact on health. Second, focusing on quantitative studies enables a structured synthesis of evidence on measurable outcomes. Every included study provides numerical results (e.g. changes in mortality rates and life expectancy) that we systematically compare and discuss.

#### Search strategy

We searched the following databases, covering the period from 1990 to March 2022: MEDLINE, EMBASE, EconLit, CINAHL, Web of Science, PsycINFO, SCOPUS, Google Scholar, Health Systems Evidence, HMIC, and ProQuest (a database for dissertations and theses). The year 1990 broadly aligns with the period when systematic reporting of DAH began, following the establishment of key global health financing mechanisms and increased availability of cross-country health data. This ensured that our review captured studies conducted in a context where DAH flows were consistently tracked and reported (Apeagyei et al. 2025). The websites and electronic libraries of key development agencies, philanthropic foundations and bilateral donors were also searched. The latter Foreign and Commonwealth Development Office, the Norwegian Agency for Development Cooperation (NORAD), the Swedish International Development Cooperation Agency, the World Bank, and the World Health Organization (WHO). Lastly, a backwards citation search of the included studies was undertaken. In PECO-S terms, the search strategy (see Table 1) was limited to exposure, comparator, and outcome terms (Higgins et al. 2019). The search strategy was also adapted to run across multiple databases to identify records within the scope of the review (see Supplementary Appendix S1 for the detailed search strategies for each database).

**Table 1:** Summary of the search strategy.

PECO-S	Searches
(search#)	
EC (1)	(Official development assistance OR official development assistance for health OR ODA) OR (development assistance for health or development assistance or external aid or foreign aid or donor resources) OR (global health initiative* or global health financ* or donor assistance or donor programmes) OR (aid adj3 (disbursement* or commitment* or flow* or international or development or project* or programme*)).tw.
**O (2)**	(mortalit* or morbidit* or death* or fatalit* or prevalence or inciden*) OR (DALYs* or life expect* or heath outcome*)).tw.
**ECO (3)**	(1) AND (2)

*Represents words with multiple endings.

#### Study selection

Records from the database search were exported to COVIDENCE, a web-based platform for supporting systematic reviews (Veritas Health Innovation 2025). Reports from websites and unpublished sources were reviewed separately. The lead reviewer (N.C.) applied the eligibility criteria to all potential studies identified through the search strategy, first screening titles and abstracts, and then conducting full-text screening. A second reviewer (F.L.) independently reviewed 25% at both the title/abstract and full-text screening stages. Three additional reviewers (M.S., S.M., and R.S.) independently reviewed the inclusion criteria and approximately 8% of the articles to ensure the consistency and reliability of the selection process.

#### Data extraction and management

Data extraction was conducted by one reviewer (N.C.), using a specifically designed template. The template was first piloted on a small sample of studies and quality checked by a second reviewer (S.M.) to assess its suitability and comprehensiveness (see Supplementary Appendix S2). A third reviewer (R.S.) validated the data extraction for all cited studies, with any disagreements resolved through discussion.

#### Risk of bias assessment

The risk of bias (RoB) assessment was conducted by one reviewer (N.C.), with a sample of studies independently assessed by 3 other reviewers each cross-validating 16% of the studies. Two tools were combined to determine the RoB of included studies (see Supplementary Appendix S3): the RoB in Non-Randomized Studies of Interventions (ROBINS-I) (Sterne et al. 2016) and the Quality of Effectiveness Estimates from Non-Randomized Studies (Faria et al. 2015). This adapted tool enabled a detailed assessment of quantitative studies, including evaluation of the steps taken to test whether the underlying model assumptions are satisfied (which were not included in ROBINS-I). For example, if a study used a DiD method, the RoB tool suggested checking whether the parallel trends assumption was tested and upheld. Supplementary Appendix S4 provides a summary of the primary analytical methods included in our search strategy, their key assumptions, and the standard tests used to assess whether those assumptions hold in each study. Overall, studies were evaluated across the following three domains: general characteristics of the study, the relevance and applicability of the method, and the estimation and reporting of the outcome. Each of these three domains was assessed for RoB and rated 1 for high RoB, 2 for moderate RoB or 3 for low RoB. The overall RoB study rating was determined by score ranking, with ranges of 0–4, 5–7, and 8–10 classified as high, moderate, and low risk of bias, respectively.

A study was deemed high quality (low RoB), if it (i) applied different methods to test the robustness of findings to alternative assumptions, (ii) thoroughly discussed the identification strategy, including the underlying assumptions, (iii) selected an appropriate model consistent with the outcome variable and adequately assessed that the identifying assumptions of the model held, either empirically or theoretically or both, (iv) considered and accounted for all important (known) confounding domains and time-varying factors to validly and reliably measure the effect, and (v) clearly defined intervention (exposure) and control groups. Lastly, the results needed to be robust in the presence of missing data, and the impact of missing data on the estimated outcomes had to be fully accounted for (Faria et al. 2015).

To further evaluate the quality of evidence, studies were graded using the Grading of Recommendations, Assessment, Development, and Evaluation (GRADE) method, a commonly used approach to appraise the quality of a body of evidence (Schünemann et al. 2020). According to GRADE, observational studies are assumed to have low certainty of evidence and are rated upwards subject to assessment. We followed this guidance and rated studies up (down) as having high, moderate, low and very low certainty in evidence, as well as their ability to account for confounding effects (Guyatt et al. 2008, 2011).

#### Data synthesis and analysis

We adopted a narrative synthesis approach (Popay et al. 2006), given the heterogeneity in study design, outcome indicators, DAH type, and the compilation of the study sample. First, we summarize the included studies, present the RoB assessments, and describe the methodological approaches used to obtain the causal estimates identified in the included studies. We then synthesize the findings by the nature of their sample—cross-country studies, pooled cross-country household-level studies, and country-specific studies—and by the primary identification strategy employed (DiD, IV, etc.). Lastly, we emphasize the findings from high-quality studies and provide evidence on the essential factors that moderate the effectiveness of health aid, to the extent this was addressed in the included studies.

## Results

### Study selection

**Figure 1:**
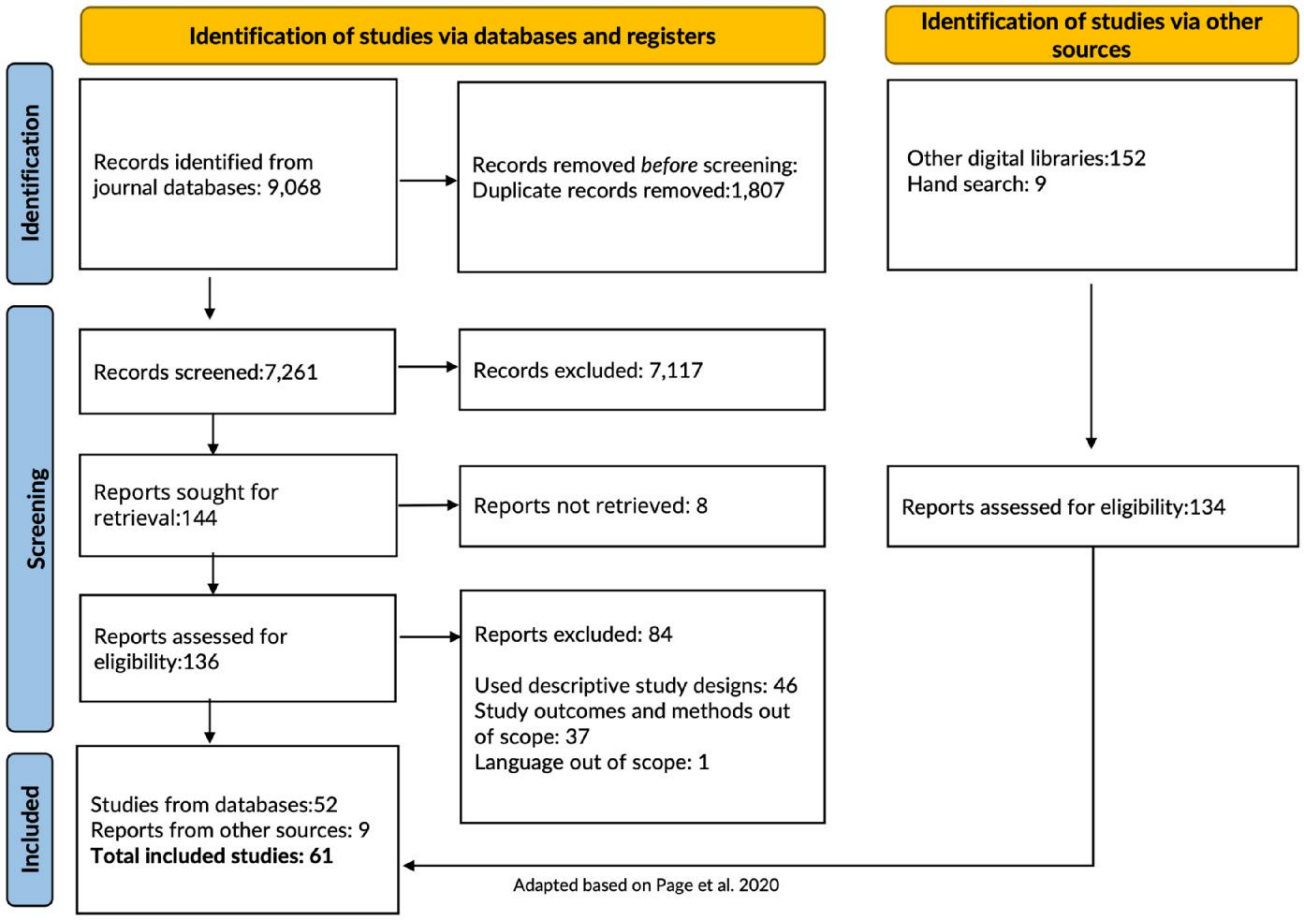
PRISMA flow statement.

Figure 1 shows the PRISMA flow of studies for this review. In total, 9068 studies were identified from the sources listed in section Study selection. After removing 1807 duplicate records, 7261 studies were screened for title and abstract. Of these, 7117 were excluded, leaving 144 studies for full-text review. Eight of these full-text reports could not be retrieved due to limited access, i.e. only abstracts were available, or articles were not accessible through the searched databases, resulting in 136 reports being assessed for eligibility. Following full-text review, 52 studies met the inclusion criteria from the main systematic search. The reasons for exclusion at this stage included applying a descriptive study design (46 studies), methods and outcomes being outside the scope (37 studies), or the study being written in a non-English language (1 study). In addition, 152 reports were identified through targeted searches of other digital libraries, and eight studies were identified through hand searches. Refer to Fig. 1, the PRISMA flow statement, which details the number of studies at each stage. Following the eligibility assessment, 9 additional studies were included, resulting in a total of 61 studies that were included.

### Publication year and study samples

**Figure 2:**
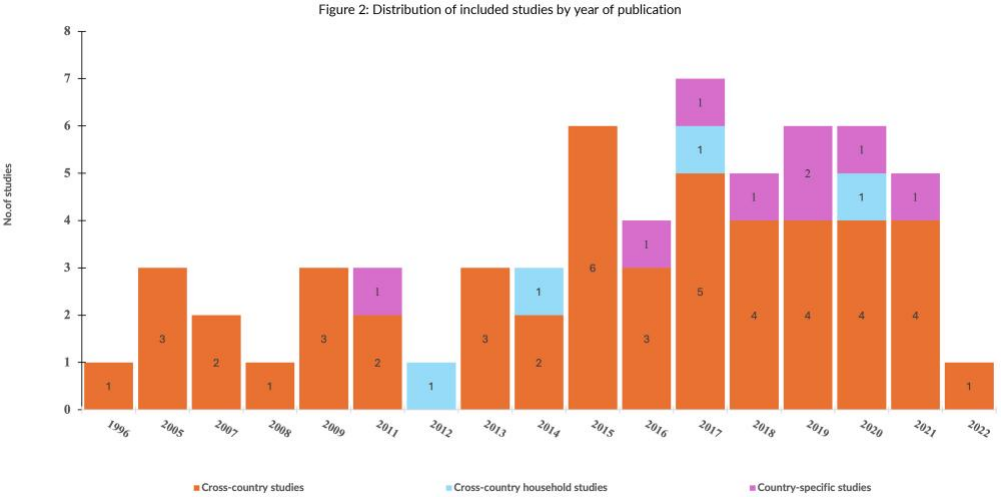
Distribution of included studies by year of publication.

Although we restricted the search to begin in 1990, the earliest eligible study was identified in 1996. Figure 2 presents the distribution of included studies by the year of publication (within the set range of 1996–2022) and country-sample type. Forty-eight of the 61 studies were cross-country macro studies, with samples ranging from 10 countries (Akinlo and Sulola 2019) to 208 (Williamson 2008). Eight studies were country-specific, examining DAH effects in Malawi, Uganda, Cote d’Ivoire, Ethiopia, Nigeria, Vietnam, India, and Zimbabwe. The other five studies were cross-country household studies (i.e. merging household-level data from several countries). Out of the included studies, 89% were published after 2010. The country sample(s) included across studies differed substantially, as did the included observation years (see Supplementary Appendix S6).

### Challenges in estimating the effects of development assistance for health

All of the included studies acknowledged concerns about endogeneity, a statistical issue that arises when the assumptions underlying a regression model fail to produce unbiased estimates. Specifically, endogeneity occurs when factors that influence both the independent variable (e.g. DAH allocation) and the dependent variable (e.g. health outcomes) are inadequately controlled for, leading to biased causal estimates (Wooldridge 2006). In the context of DAH programming, endogeneity arises from the non-random nature of aid distribution, which is shaped by political, strategic, and diplomatic considerations, as well as by relationships between donors and recipient countries (Akinlo and Sulola 2019, Pickbourn and Ndikumana 2019). Countries receiving DAH may differ systematically from those that do not, creating potential selection bias (Lee and Izama 2015). Moreover, a bi-directional relationship between DAH flows and health outcomes complicates causal inference, as a country’s health status may influence the amount of aid it receives. In contrast, aid itself can impact health outcomes, creating a feedback loop that makes it difficult to estimate the actual effect of DAH on health (Kim 2018).

**Figure 3:**
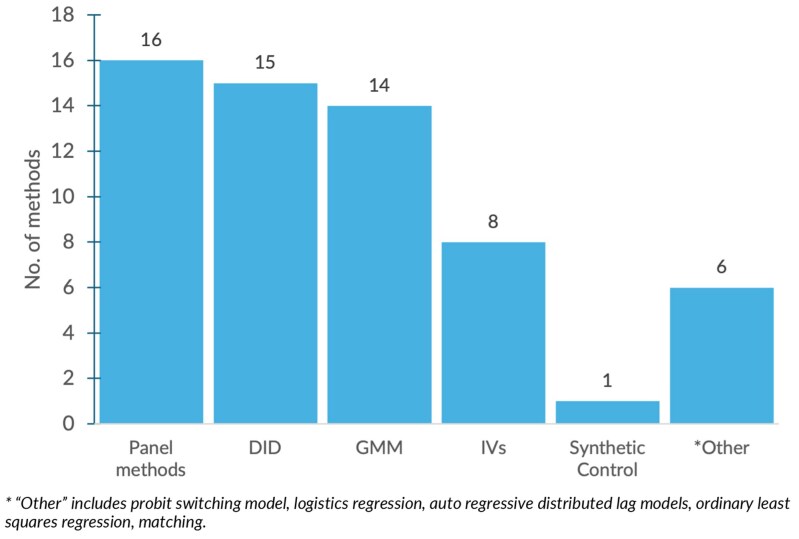
Summary of primary estimation methods.

It is also important to recognize that accounting for all the factors influencing aid effectiveness in empirical analyses is highly challenging, as many of these factors may be unobserved or unavailable in the data. These limitations can lead to biased estimates (Gyimah-Brempong 2015, Yogo and Mallaye 2015, Jaupart et al. 2019). To address endogeneity, the studies included in this review employed various statistical methods to analyse the effects of health aid on health outcomes. However, the rigour of these approaches differed (Burnside and Dollar 2009, Mishra and Newhouse 2009, Burguet and Soto 2013, Pallas and Ruger 2017, Hein et al. 2020). Supplementary Appendix S4 describes the methods employed across the included studies, the underlying data assumptions of each method, and the common approaches to testing these assumptions. As detailed in the Risk of bias assessment section in Methods, studies rated as high quality employed at least two identification methods to test the robustness of their findings. Figure 3 summarizes the primary statistical methods used across the studies.

Another concern in this research is the reliability of the data used. DAH estimates are typically derived from multiple sources, increasing the likelihood of measurement error (Afridi and Ventelou 2013). The OECD and Institute for Health Metrics and Evaluation (IHME) estimates of DAH, for instance, rely on combined data from various organizations, which may introduce measurement errors (IHME 2020). Furthermore, country-specific studies often rely on population-based household surveys, such as the Demographic and Health Surveys (DHS), to estimate disease burden, including mortality rates. These surveys typically ask whether households (respondents) have experienced episodes of morbidity (illness) or encountered mortality of a household member within a given period preceding the survey. This method is prone to recall bias, which could potentially skew causal estimates (Marty et al. 2017, Odokonyero et al. 2018).

Taken together, these issues can affect the validity of findings regarding the estimated relationship between DAH and health. However, a range of statistical/econometric methods exists to address or at least mitigate these threats to study validity. These methods can be broadly grouped into two main categories of (i) selection on unobservables, e.g. difference-in-difference, IVs, synthetic control, and generalized methods of moments, and (ii) selection on observables, e.g. switching probit model and auto-regressive distributed lag model (Angrist and Pischke 2009, Jones et al. 2013). The identifying assumptions for each category are outlined in Supplementary Appendix S4. In this review, 23 of the included studies sought to address these issues by applying at least 2 estimation methods in their analyses. They commonly presented a ‘primary method’ alongside one or more alternatives as a robustness check of the validity and reliability of the findings.

### Quality assessment of studies

The 61 included studies were rated as high (*n* = 22), moderate (*n* = 25), or low (*n* = 14) quality, based on the RoB criteria set out in the Methods Risk of bias assessment section. Focussing on the high-quality studies, eight applied DiD as their ‘primary method’, seven employed GMM, five used IVs, three utilized panel methods and one was based on synthetic control methods to estimate causal estimates. As per our RoB criteria, all applied at least one alternative econometric approach as a robustness check. The high-quality rated studies are presented in Table 2, and a complete list of included studies is given in Supplementary Appendix S6. The health impact estimates of all included studies are described in the section Health outcomes investigated, along with additional summaries from the high-quality studies.

**Table 2:** Extract of high-quality studies and direction of association of key variables.

		Boone (1996)	Williamson (2008)	Williamson (2008)	Mishra and Newhouse (2009)	Akachi and Atum (2011)	Wagstaff (2011)	Wilson (2011)	Wilson (2011)	Wilson (2011)	Afridi and Ventelou (2013)	Chauvet et al.(2013)	Chauvet et al. (2013)	Gyimah-Brempong (2015)	Gyima- Brempong (2015)	Gyima- Brempong (2015)	Lee and Izama (2015)	Lee and Izama (2015)	Ziesmer (2016)	Doucouliagos etal (2021)
**Scope of variables**	**Number of countries**	123	208	208	118	34	61	96	96	96	109	84	84	48	48	48	37	37	65	96
	**Overall effect**	None	None	None	Pos	Pos	Pos	None	None	None	Pos	Pos	Pos	Pos	Pos	Pos	None	None	Pos	Pos
	**RoB assessment**	High	Low	Low	Low	Low	Low	Low	Low	Low	Low	Low	Low	Low	Low	Low	Low	Low	Low	Low
	**Health outcome of interest**	IMR	IMR	MMR	IMR	CMR	CMR	IMR	CMR	Life exp	Adult mort	CMR	IMR	MMR	CMR	Cholera Mort	NMR	HIV Prev	Life exp	IMR
**Structural**	GDP		▴		▴	▴	▴	▴	▴	▴	▴	▴	▴	▴	▴	▴	●	●		
	GDPpc/ wealth						▴				▴								▴	▴
	Urban residency		▴	●													●	●		
	Education													▴	▴	▴				▴
	Mother education											●	●							
	Pop. Density				●			▴	▴	▴							▾	▾		
	Pop. Growth/ fertility	●												▾	▾	▾				▴
**Political and governance**	Governance/polity																▴	▴		▴
	Aid volatility																			
	Freedom Index		▴	▴																
	Donor multiplicity																			
	Corruption control																			
	Government effectiveness																			
	GNIpc																			
	DAHpc/ ODA	●	●	●	▴	▴	▴	▴	▴	▴	▴	▴	▴	▴	▴	▴	◄►	◄►	▴	▴
**Health system related**	HIV				▾	▾														
	HRH density		▴	▴	●	●	●	●	●	●	●	●	●	●	●	●				●
	Immunization coverage																			
	Public health expenditure						▴				▴									
	Health aid fragmentation																			
	OOP spending						▴				▴									
**Biological and behavioural**	Sanitation					▴														
	Mosquito net ownership					▴														
	Parity																			
	Obesity																			
	Prenatal visits																			
	Body Mass Index																			
	Drinking water					▴														
	Safe drinking					▴														

		Pallas and Ruger (2017)	Marty et al. (2017)	Yogo and Mallaye (2015)	Yogo and Mallaye (2015)	Yogo and Mallaye (2015)	Akinlo and Sulola (2019)	Akinlo and Sulola (2019)	Jaupart et al. (2019)	Jaupart et al (2019)	Martorano et al. (2020)	Martorano et al. (2020)	Cruzatti et al. (2023)	Woode et al. (2021)	Fichera et al. (2021)	Fichera et al. (2021)	Fichera et al. (2021)	Weiss et al. (2022)
	**Number of countries**	139	1	33	33	33	10	10	84	84	13	13	53	68	1	1	1	25
	**Overall effect**	Pos	Pos	Pos	Pos	Pos	Pos	Pos	Pos	Pos	Pos	None	Pos	Pos	Pos	None	None	Pos
	**RoB assessment**	Low	Low	Low	Low	Low	Low	Low	Low	Low	Low	Low	Low	Low	Low	Low	Low	Low
	Health outcome of interest	IMR	Malaria prev	HIV Prev	CMR	IMR	IMR	CMR	CMR	IMR	CMR	Stunt	IMR	IMR	CMR	IMR	NMR	CMR
**Structural**	GDP																	
	GDPpc/wealth	▴	▴	●	●	●	▴	▴						▴	●	▴	●	
	Urban residency			●	▴	●												
	Education														▴	●	●	
	Mother education				▴	●												
	Pop. Density			▴	▴	●								▾				
	Pop. Growth/fertility																	
**Political and governance**	Governance/polity					●												
	Aid volatility																	
	Freedom Index	▴																
	Donor multiplicity																	
	Corruption control													●				
	Government effectiveness													▴				
	GNIpc																	
	DAHpc/ODA	▴	▴	●			▴	▴	▴	▴	▴	▴	▴	●	▴	●	●	▴
**Health system related**	HIV													▾				
	HRH density																	
	Immunization coverage						▴	▴										
	Public health expenditure			●	●	●	●	●										
	Health aid fragmentation																	
	OOP spending																	
	Safe drinking																	

▴ Positive effect; ▾ negative effect; ● no effect (none); ◄► mixed findings.

RoB, risk of bias; IMR, infant mortality rate; CMR, child (under-5) mortality rate; MMR, maternal mortality rate; NMR, neonatal mortality rate; Life exp, life expectancy; HIV Prev, HIV prevalence; GDPpc, gross domestic product per capita; GNIpc, gross national income per capita; DAHpc, development assistance for health per capita; ODA, official development assistance; HRH, human resources for health; OOP, out-of-pocket; etc.

### Health outcomes investigated

A total of 117 health outcomes were examined across the studies, with considerable overlap. Thirty-seven studies focused on a single outcome, while the rest investigated multiple outcomes (up to 14). Mortality outcomes were prominent (69%, *n* = 81), followed by disease-specific incidence and prevalence indicators and DALYs (19%, *n* = 22), and life expectancy (12%, *n* = 14). Maternal, neonatal and child health (MNCH) related mortality and morbidity indicators were most common (55%, *n* = 64) and distributed as follows: maternal mortality rate (MMR = 3/64), infant mortality rate (IMR = 25/64), under-five-mortality rate (31/64), neonatal mortality (within 28 days) 4/64, and neonatal fever (1/64).

**Figure 4:**
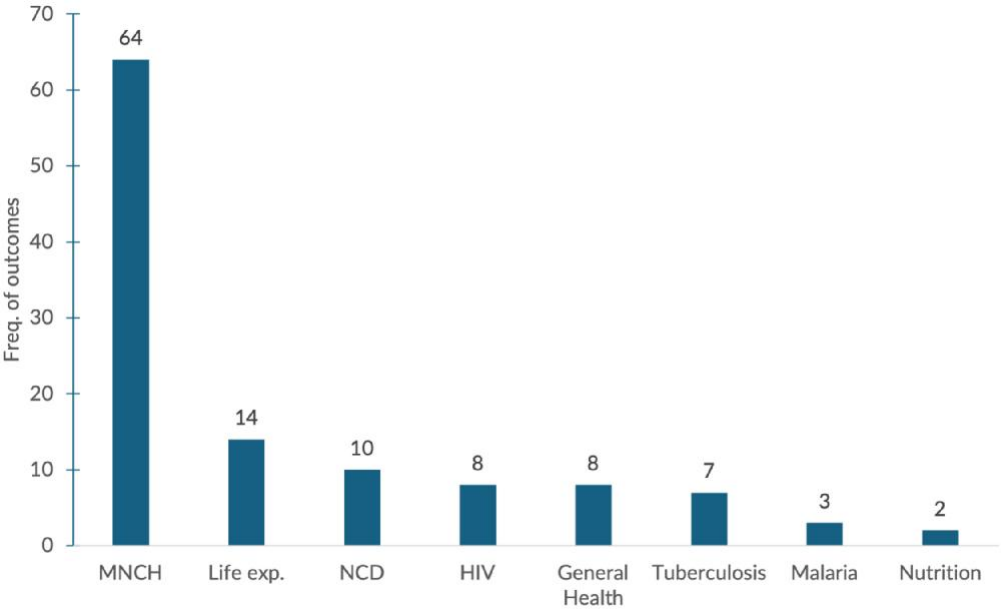
Summary of reported health outcomes by disease areas.

The dominance of MNCH outcomes is not surprising, as they are widely viewed as responsive to changes in the quality of primary health care, and hence arguably well-suited for assessing the effects of DAH on population health (Boone 1996, Bhaumik 2005, Burnside and Dollar 2009, Weiss et al. 2022). It may also be because they are most frequently measured in commonly used surveys, including the DHS (which may reflect the particular attention they have long received in global health programmes). Thirteen studies investigated other disease-specific indicators, including Non-Communicable Diseases (NCD)-related DALYs, tuberculosis, malaria and Human Immuno Virus (HIV). Ten studies examined life expectancy. Figure 4 summarizes the different types of health outcomes investigated.

### Findings regarding the impact of development assistance for health on health outcomes

#### Cross-country studies

The 48 cross-country studies investigated 100 out of 117 (85%) different health outcomes, with 54% (54/100) focusing on maternal, newborn, and child health (MNCH). Out of these studies, 32 reported significant positive effects of DAH (i.e. health-improving), one reported a negative effect, seven showed no effects, and eight others indicated a mix of positive and insignificant impact across different outcomes. Of the 48 studies, eighteen were assessed as ‘high quality’, out of which 13 found a positive impact of health aid on health, while four found no evidence of effect. The remaining study reported no effect of health aid except when conditioned on governance. Another 19 studies were classified as ‘moderate quality’, with seven emphasizing a health-improving effect of DAH, two stating no effect, and one reporting a negative effect. The remainder (*n* = 11) studies were deemed ‘low quality’.

Turning to a summary of the statistical methods used in the included studies, 15 employed panel econometric methods. Of these, 11 studies exclusively identified positive effects (quasi-experimental or highly likely to be causal) of DAH on life expectancy (Bendavid and Bhattacharya 2014, Gillanders 2016), as well as on infant and child mortality (Pettersson 2007, Akachi and Atuni 2011, Chauvet et al. 2013, Bendavid and Bhattacharya 2014, Pickbourn and Ndikumana 2019). Four studies yielded conflicting results across the multiple outcomes each investigated (with both no effect and positive, i.e. health-improving effects) on infant, child & adult mortality rates (Yan et al. 2015, Toseef et al. 2019), infectious disease-specific mortality rates [positive impact on HIV and malaria, no effect on Tuberculosis (TB)] (Hsiao and Emdin 2015), and Non-Communicable Diseases (NCD) related mortality and morbidity (Kostova et al. 2021). For example, Yan et al. (2015) estimated that a $10 per capita increase in Global Fund disbursements reduced all-cause adult mortality by 1.4% and malaria-specific under-five mortality by 6.9% but did not significantly increase all-cause under-five mortality. The positive effect sizes varied across studies, as did the composition of the study samples. The average estimated reductions in infant mortality for a 1% increase in DAH ranged from 0.14% (Pettersson 2007) to 2.6% (Negeri and Halemariam 2016). For life expectancy, Bendavid and Bhattacharya (2014) found that DAH increased life expectancy by 0.24 months, equivalent to a 0.03% increase relative to a baseline of 70 years. Similarly, Gillanders (2016) reported a 0.02% increase in life expectancy, as a proxy for human development, in response to positive aid shocks in democratic settings.

Six studies utilized DiD designs as their primary method. Three found statistically significant positive effects: for MNCH outcomes (Bhaumik 2005, Jaupart et al. 2019) and adult mortality in general (Hein et al. 2020). The other three studies investigated the effectiveness of different modes of delivering health aid. Woode et al. (2021) found that implementing a sector wide approach (SWAp) to coordinate health aid delivery significantly reduced infant mortality compared to the counterfactual of comparable (propensity-matched) non-implementing countries. Kim (2018) found that implementing a ‘President’s Emergency Plan for AIDS Relief’ (PEPFAR) delivery mechanism significantly reduced HIV transmission and mortality in women and children. However, Lee and Izama (2015) found that the siloed PEPFAR approach yielded unintended effect, slowed down the rate change, on all-cause neonatal mortality. Closely related to DiD design, Weiss et al. (2022) applied a SCM to estimate the impact of United States Agency for International Development (USAID)-funded child health programmes on child mortality, finding that higher-than-average USAID funding allocations were associated with 29 fewer under-five deaths per 1000 live births compared to the counterfactual. Although both DiD and SCM are widely used in policy evaluations, they have limitations, including capturing treatment effect heterogeneity, selection bias in donor pools, and potential spill-over effects. The RoB assessment was vital in mitigating these risks, enabling the synthesis of findings from high-quality studies.

Fifteen studies used dynamic panel (GMM) methods as their primary approach, accounting for country fixed effects to examine various health outcomes, including MNCH, cholera mortality, TB, HIV incidence, and life expectancy. Of these 15, nine studies exclusively found positive effects on MNCH outcomes (e.g. Masud and Yontcheva 2005, Mishra and Newhouse 2009—IMR), adult mortality and life expectancy (e.g. Afridi and Ventelou 2013), and HIV reduction (e.g. Yogo and Mallaye 2015)—IMR. Han and Koenig-Archibugi (2015) emphasized that, up to a point, having more development partners in the health sector is better than having fewer ones, in terms of improving child mortality rates. Four studies utilizing GMM identified no significant effects of DAH on health outcomes. Wilson (2011) highlighted their GMM results (among numerous estimation methods), showing that health aid did not affect infant and child mortality or life expectancy, and instead noted that donors tended to send more aid to countries that had experienced greater reductions in IMR. Similarly, Pallas and Ruger (2017) found that neither general health aid nor reproductive health aid influenced infant mortality. Tarverdi and Rammohan (2017) found no impact of health aid on child mortality except when it interacted with ‘good’ governance. Their study utilized a composite index to represent governance, derived from the six World Governance Indicators: voice and accountability, political stability and absence of violence, government effectiveness, regulatory quality, rule of law, and control of corruption, as documented. This finding was supported by Burguet and Soto (2013), who found no overall effect of health aid on child mortality, except for sub-sector health aid (aid for basic health and population programmes). The study found that a US$1 per capita increase in basic health expenditure was associated with a 1.9% decrease in child mortality per 1000 live births.

Other GMM studies found that DAH did not yield any positive effect on reducing TB incidence (Mukherjee and Kizhakethalackal 2013) or HIV prevalence (Delacruz 2018). Patenaude (2021) found mixed results: a US$1 increase in DAH for HIV and malaria reduced under-five mortality by 0.002% and malaria incidence by 0.018%, respectively, when channelled through government systems, but had no effect when delivered via non-governmental systems. Oryema et al (2017) found that countries that benefited from debt relief experienced a 4.72% reduction in child mortality relative to similar non-beneficiary countries. In contrast to other GMM studies, Nwude et al. (2020) found adverse effects of DAH: a 1% increase in aid was associated with a 0.04% rise in under-five mortality and a 0.01% decrease in life expectancy. While GMM is designed to handle endogeneity, the choice of instruments, including finite sample bias, can lead to inconsistent estimates.

Seven studies used IVs to assess the impact of DAH on MNCH outcomes and on health outcomes related to fever and diarrhoea. Four of these studies exclusively found positive effects on disease prevalence and infant and child mortality (Burguet and Soto 2013, Chauvet et al. 2013, Cruzatti et al. 2023). Specifically, Cruzatti et al. (2023) observe that a Chinese aid project implemented in Sub-Saharan Africa averted 10 infant deaths for every 1000 live births. Doucouliagos et al. (2021) found no overall effect on infant mortality but did find a significant interaction with good governance: a 1% increase in health aid under good governance resulted in a 0.034%–0.047% reduction in infant mortality. Similarly, Burnside and Dollar (2009) found that in countries with good governance, an additional 1% of GDP in aid was associated with a 0.9% decrease in infant mortality. However, Williamson (2008) found that DAH had no impact on infant or child mortality. Apart from challenges with instrument validity, IV findings apply only to the studied (sub)population, limiting their generalizability to other settings.

Other studies (Gomanee et al. 2005, Mukherjee and Kizhakethalackal 2013) utilized quantile regression to explore the effects of DAH on infant mortality. The former used a sample of 110 countries and found that DAH was effective in countries at lower IMR quantiles but was not effective in countries with higher IMRs. The latter reported that an additional 1% of aid led to a 0.5% decrease in infant mortality across all quantiles. However, in their sample of 38 countries, the impact was relatively larger for countries below the median welfare distribution (i.e. those with lower Human Development Index scores). An influential study by Boone (1996) was one of the first to examine the impact of aid on social outcomes, including infant mortality and life expectancy. The study employed panel fixed effects and IV methods, suggesting that long-term aid has had no impact on health outcomes and other social indicators. However, the study highlighted that health outcomes were influenced by the nature of political regimes, as liberal and democratic settings showed an average decline of 30% in infant mortality compared to autocratic ones, suggesting that aid might be more effective if targeted at supporting new liberal regimes.

In other estimation approaches, Wolf (2007) applied 2SLS to investigate the effect of aid on MNCH outcomes, finding no significant impact on infant mortality but a positive effect of 0.14%–0.17% on under-five mortality. Herzer (2019) applied an autoregressive distributed lag dynamic panel approach to investigate the long-term effect of health aid. The findings showed temporary reductions in infant mortality; however, in the long run, health aid increased infant mortality by 0.179 per 1000 live births. The variations observed across countries seemed to be at least partially driven by the political environment. Liberal regimes appeared to promote policies that benefit the poor and improve institutional quality (Wolf 2007). This suggests that effective measures to combat corruption can have significant positive effects on infant mortality and other development indicators. Although some of the methods applied in the studies are less reliable in resolving concerns about endogeneity, selection bias, and measurement errors, they still show some marginal health-improving effects of DAH.

#### Pooled cross-country household-level studies

Five studies aggregated cross-country household-level data, with three applying a DiD design, one utilizing panel-fixed effects, and the remaining study applying Coarsen exact matching. Among these studies, Martorano et al. (2020) was rated as high quality (low risk of bias). This study examined the impact of receiving Chinese aid on child mortality and stunting in 13 African countries, finding significant reductions in child mortality (0.9%) but no significant effect on stunting. However, the impact on stunting observed in the prior study contrasts with Rustad et al. (2020), who found that exposure to World Bank-sponsored aid projects significantly improved the nutritional status of children exposed to drought. The other three studies each reported significant positive effects of DAH on health outcomes. Bendavid et al. (2012) examined the effectiveness of PEPFAR's aid delivery model, finding that adult mortality fell from 8.3 to 4.1 deaths per 1000 adults in PEPFAR focus countries between 2003 and 2008. In contrast, PEPFAR non-focus countries experienced a smaller reduction from 8.5 to 6.9 deaths per 1000 adult population over the same period (OR = 0.86, *P* = 0.03).

More recently, Bendavid (2014) found that health aid was both effective and pro-poor: a $1 increase in per capita DAH was associated with 0.57 fewer child deaths per 1000 person-years in the poorest quintile compared to the wealthiest quintile. Further, they found that malaria-specific health aid was particularly beneficial for the poor. Finally, Jakubowski et al. (2017) reported that receiving USAID funding through the President Malaria Initiative (PMI) reduced the under-five mortality rate from 28.9 to 24.3 per 1000 person-years compared to the period before the PMI mechanism. The effect was larger among children with more educated mothers and those living in wealthier households and urban areas.

#### Country-specific studies

Seven of the eight studies reported exclusively positive effects of DAH, including three that were high-quality. One study, also rated as high quality, found mixed effects (Fichera et al. 2021). Four of the eight studies explored the spatial effects of health aid on health outcomes. For example, Odokonyero et al. (2018) analysed the impact of aid-funded projects on self-reported disease burden and days of lost productivity due to ill-health by linking aid project data with household surveys in Uganda, and using a spatial DiD approach. Their findings indicated that increased health aid disbursements reduced the self-reported disease burden among households living within 15 km of funded projects, though the evidence was weak. However, there was stronger evidence that increased aid disbursement reduced days of lost productivity by 52%–70% for a 1% increase in aid disbursements.

Kotsdam et al. (2018) also assessed the importance of proximity to aid projects, finding that living within 25 km of an active aid project in Nigeria reduced the incidence of infant mortality by 2.6%. Similarly, Marty et al. (2017) found that living in a ‘traditional authority’ (i.e. a sub-district area) in Malawi that received infrastructure or parasitic control aid reduced malaria prevalence by 1.4% and 2.2% points, respectively. Wayoro and Ndikumana (2020) found that living within 50 km of an aid project in Cote d'Ivoire reduced infant mortality by approximately 2%–3%, particularly for water and sanitation projects. Though not a spatial analysis, Gutema and Mariam (2018) also found evidence of aid effectiveness in Ethiopia, observing that a 1% increase in DAH increased life expectancy by 0.026 years (9.5 days) in the year immediately following aid disbursement, and by 0.008 years (3 days) two years post-provision of assistance.

In DAH programming, fungibility—the tendency for recipient countries to utilize aid for purposes unrelated to its intended scope—continues to be a significant cause for concern (Dieleman et al. 2013, Van de Sijpe 2013). Wagstaff (2011) investigated the effect of fungibility by comparing changes in health outcomes before and after aid flow in 61 provinces in Vietnam, accounting for intersectoral fungibility between project and non-project areas. The study reported positive effects, with aid reducing under-five mortality by 25%–27%, even after accounting for fungibility effects. The reported effects were also proportional to the productivity of government spending, which was assessed as being similar in both project and non-project areas.

Two country-specific studies, conducted in India and Zimbabwe, equally provide context-specific insights into the effect of DAH. In the former, an evaluation of NORAD funding for maternal and child health interventions in India observed that the programme significantly reduced neonatal fever and post-discharge mortality by 17.6% and 2.6%, respectively (NORAD 2018). In a later study, Fichera et al. (2021) analysed the impact of the results-based financing (RBF) model on MNCH health outcomes in Zimbabwe. RBF allocates funds to service delivery units or health programmes based on the achievement of predetermined results or outcomes. In this context, the study found that RBF resulted in a 2% reduction in under-five mortality but had no significant effects on infant or neonatal mortality.

### Mechanisms of impact and subgroup effects

Thirty-three of the studies reported that the impact of DAH was relatively greater in countries with higher per capita GDP (Bhaumik 2005, Gomanee et al. 2005), higher literacy levels (Fichera et al. 2021), and stronger governance systems (Burnside and Dollar, 2009, Tarvedi and Rammhan 2019). Thirteen of the 33 studies examined causal pathways from DAH to health outcomes, while the remaining studies applied control variables without considering channel-specific mechanisms. The 13 studies can be broadly classified into those exploring spatial mechanisms, DAH delivery channels, governance systems, structural factors, and programme-specific drivers (see Supplementary Appendix S7).

#### Proximity to development assistance for health-funded projects

Four country-specific studies (in Malawi, Nigeria, Uganda, and Cote d’Ivoire) examined the role of geographic proximity to DAH–funded projects in enhancing aid effectiveness for health (Marty et al. 2017, Kotsdam et al. 2018, Odokonyero et al. 2018, Wayoro and Ndikumana 2020). Across these studies, healthcare utilization and consequent health outcomes were found to be more favourable among communities and individuals residing closer to aid projects, particularly those related to health facilities, schools, and roads. Similarly, Rustad et al. (2020) found a positive effect of historical aid on the nutrition status of children exposed to drought-related aid initiatives funded by the World Bank. This suggests that increasing accessibility to critical infrastructure and social amenities through proximal zoning can be a key (and plausible) pathway to enhancing aid effectiveness. Supplementary Appendix S7 highlights studies that examined the mechanisms of these effects.

#### Vertical (disease-specific) funding

Vertical (or siloed) funding can help improve related health outcomes, but can also create negative externalities. Kostova et al. (2021) found that vertical funding for NCDs appeared to have short-term positive effects on NCD-related outcomes. Similarly, Bendavid et al. (2012) and Kim (2018) found that adult mortality and HIV infection rates were substantially lower in PEPFAR-supported countries. However, Lee and Izama (2015) hypothesized that such vertical funding approaches can have unintended negative effects. Testing this, they found that, on average, PEPFAR mechanisms had a small deleterious effect, slowing the rate of reduction in neonatal mortality by 0.6%. The study suggested that such unintended consequences in aid programming are difficult to avoid. They further noted that these negative effects tend to be driven by supply-side factors, such as crowding-out, internal brain drain, and absenteeism and reallocation effects, and underscored that such targeted aid programmes can weaken state capacity.

#### Delivery via government versus non-government channels

Patenaude (2021) indicated that aid provided through government systems yielded greater system-wide benefits and sustained more substantial average reductions in under-five mortality, TB incidence, as well as increases in life expectancy, compared to aid provided via non-governmental channels such as NGOs. They also found that HIV-DAH reduced under-five mortality rates when delivered through on-budget methods (via recipient government systems), but not when delivered via off-budget channels. In contrast, however, a study involving 58 countries found that official bilateral aid (often largely on-budget) had no significant impact on reducing infant mortality. In comparison, NGO aid did (Masud and Yontcheva 2005). The study noted that, in contrast to NGO aid, on-budget DAH tended to be trapped in bureaucratic processes and to trickle down only partially to grassroots levels.

Building on this, Kostova et al. (2021) provide further nuance by distinguishing between vertical aid (earmarked for specific diseases) and horizontal aid (channelled through public systems to strengthen broader health sector capacity). Their study found that vertical aid produced immediate and measurable reductions in NCD mortality and morbidity, while horizontal aid did not show short-term effects on these outcomes. However, horizontal aid was linked to delayed but significant improvements in key risk factors, such as reductions in elevated blood pressure. This suggests that channelling resources through public systems may be slower to produce visible health gains. Still, it plays a critical role in building long-term health system capacity, supporting sustainable improvements in service delivery and chronic disease management.

#### Good governance and public systems

Several studies examined the role of governance, including government effectiveness in achieving health impacts from aid (Pettersson 2007, Burnside and Dollar 2009, Tarverdi and Rammohan 2017). Together, these studies suggest that political systems can play a crucial role in reshaping the effectiveness of health aid—when used efficiently and aligned to recipient countries’ priorities and needs. For example, Boone (1996) found that aid overall was ineffective; however, countries with democratic regimes or those undergoing democratic reforms experienced decreases of up to 30% in infant mortality, in contrast to autocratic regimes.

#### Programme-specific drivers and public spending

Programmatic factors have also been found to influence the impact of DAH. The scale-up of malaria prevention technologies—namely, IRS, ITNs, and artemisinin-based combination therapies—significantly reduced the burden of all-cause child mortality (Akachi and Atun 2011, Jakubowski et al. 2017). Some studies found that the effects were contingent on how malaria affected under-five mortality, and that prioritizing aid to such settings could avert more deaths. Yogo and Mallaye (2015) separated the direct and indirect effects of public health spending on health outcomes. This pathway appeared not to yield impact until after the Millenium Development Goals were launched. The study suggested that 37% of the total aid effects on child mortality were mediated by public health expenditure, clearly demonstrating the relevance of deliberate policies to improve domestic general government spending, which has a far-reaching impact on population health.

#### Education and health infrastructure

Within-country analyses indicate that, at the household level, health aid benefits families with higher maternal literacy and those in the upper wealth quintile more significantly (Fichera et al. 2021). The link between maternal literacy and health outcomes highlights the critical role of female education, especially in LMICs. In line with this, Yogo and Mallaye (2015) separated the direct and indirect effects of education on health outcomes. The study suggested that 64% of the total aid effects on child mortality were mediated by female education. Educated mothers are more likely to seek healthcare, adopt healthier practices, and ensure better health outcomes for their children, thereby creating a long-term cycle of improved health.

Similarly, investments in targeted basic health infrastructure (Abraham and Tao 2021) have proven to be an effective channel for aid delivery. Accessible social amenities reduce barriers, such as transport costs and long waiting times, and, in turn, facilitate the uptake of health services. Together, investments in education and health infrastructure not only improve immediate health outcomes but also empower communities and households with the resources and knowledge to maintain better health in the long run.

#### Increasing recipient country responsibility and ownership of development assistance for health

Woode et al. (2021) evaluated the effectiveness of the Sector Wide Approach (SWAp) for aid delivery in 68 LMICs. The SWAp is a delivery mechanism for aid that broadly reflects the Paris Principles of Aid Effectiveness. The study found that implementing a SWAp significantly reduced infant mortality rates by between 5.8% and 8.1%, with the reduction occurring as the SWAp matured. The estimated effect was perceived to be driven by reduced transaction costs resulting from less fragmented aid and by leveraging development partner support to enhance the efficiency of domestic health resource utilization.

The use of the RBF mechanism has also gained popularity among donors and partners in different contexts. Through this mechanism, implementing agencies are contracted to provide services and are only paid conditionally on the attainment of objectively verified, agreed-upon performance targets. Fichera et al. (2021)found mixed results of RBF in Zimbabwe, in that it significantly reduced child mortality, but not infant and neonatal mortality. This finding suggests that while RBF programmes are complex and context-specific, they may offer a viable option for health system reform. By carefully considering the design and implementation of RBF programmes with a focus on strengthening primary health systems, there is potential for significant improvements both in the productivity of the health system and subsequent population health outcomes.

## Discussion

### General findings

DAH is a significant source of revenue for financing healthcare in LMICs, although its effectiveness has long been debated (Boone 1996, Bhaumik 2005, Marty et al. 2017). The intricate bi-directional relationship between DAH allocations and health outcomes, as well as challenges in empirically establishing causal effects, have led econometric methods to become a key aspect of the literature (Stuckler et al. 2013). While a minority of studies have found no effect or even a negative impact of health aid on specific health outcomes, the bulk of the evidence identified in this review, including that from high-quality studies, suggests that aid has improved health across a range of outcomes. Further, there are also variations in the magnitude of effects, even among studies investigating the impact of DAH on the same result, such as infant mortality rates. It appears that the composition of the sample (varying country and year compositions of datasets), the choice of health outcome(s), and the estimation methods utilized in the analyses account for much of these differences.

The international development community and policymakers may take heart that DAH has had a significant and positive impact on health outcomes in LMICs. Focusing on the studies assessed as high quality (i.e. having a low risk of bias), nineteen of the 22 studies reported significant positive effects (Mishra and Newhouse 2009, Wagstaff 2011, Afridi and Ventelou 2013, Yogo and Mallaye 2015). The four remaining high-quality studies still offer important insights. For instance, Burguet and Soto (2013) found that while aid in general did not have significant effects, aid specifically targeted at basic healthcare, population and reproductive health programmes, as well as food aid, reduced child mortality. The combined effect of these specific streams of health aid was even greater. It can be suggestive of the need to enhance donor and inter-programme (sector) coordination to maximize the effect of aid in recipient countries. Williamson (2008) found that health aid was ineffective but found positive influences of physician density on health outcomes, which may reflect the necessity for broader and more focused investments in other essential components of the health system, such as human resources for health. While DelaCruz (2018) found no significant effect of aid on HIV burden, they noted that weak surveillance systems posed considerable challenges in quantifying the impact of aid. Notably, Fichera et al. (2021) also observed that aid significantly reduced under-five mortality. However, it did not decrease infant or neonatal mortality, demonstrating that the assessment findings on the impact of DAH vary depending on the outcome used.

Further, the reviewed studies reveal that DAH has played a crucial role in supporting disease control initiatives, especially those aimed at tackling HIV, tuberculosis, and malaria (Lee and Izama 2015, Jakubowski et al. 2017, Kim 2018). For example, Yan et al. (2015) observed that an increase in per capita DAH disbursement in the later years of the Global Fund had a substantial impact on reducing adult mortality. Similarly, the impact of PEPFAR programmes on HIV pandemic control has consistently been credited with positive effects (Weiss et al. 2022). There appears to be good reason to believe that some countries are on track to attain global HIV reduction targets, and DAH has been a contributor (Yan et al. 2015, Kim et al. 2018, Patenaude 2021). The same may hold for other disease programmes, such as MNCH conditions (Akachi and Atun 2011; Wagstaff 2011).

The general finding that health aid has primarily produced health-improving effects in recipient settings is consistent with an earlier evidence. In particular, Taylor et al. (2013) reported that DAH targeting maternal and child health interventions was linked to some modest gains in health outcome. The authors also sought to assess the impact of aid allocated in accordance with the Paris Declaration and the aid effectiveness agenda, but concluded the evidence at the time did not allow such an assessment. CHD (2025) also found that DAH has a modest effect on aggregate outcomes such as infant and child mortality, with more mixed evidence on disease-specific indicators. Although the present review was not designed to specifically evaluate the comparative effectiveness of aid delivered in accordance with the Paris Principles, it is worth noting that most of the studies that reported a lack of any effect analysed data entirely or mainly from the period before the Paris Declaration, when aid and health aid was commonly criticized for being ineffectively disbursed and managed (Buse and Walt 1997, OECD 2005). See Supplementary Appendix S6 for the distribution of studies and subsequent outcomes before and after the Paris aid effectiveness agenda. These studies might therefore reflect outcomes from aid more likely to have been delivered ‘ineffectively’ (e.g. Boone 1996, OECD 2005, Williamson 2008, Kizhakethalackal 2009, Wilson 2011). Conversely, most recent studies with a larger proportion of observations post-Paris Declaration have found small positive effects of health aid. Further, Woode et al. (2021) found that implementing a sector-wide approach (SWAp) to deliver and manage health aid, in line with the Paris Principles (harmonization, alignment, and recipient country ownership), resulted in improved health outcomes. These and other findings offer some tentative evidence that aid effectiveness principles may have had some positive impact on health aid effectiveness.

Global health programming involves a multitude of agencies with heterogeneous interests. Effective coordination among these agencies may be the basis for leveraging synergies and enhancing stakeholders’ responsiveness through competitiveness, thereby improving global health. Whilst Han and Koenig-Archibugi (2015) demonstrated that despite fragmentation and proliferation of partners, health aid reduced child mortality, governments in LMICs need to develop a strong policy framework to coordinate donor efforts and in the process minimize the transaction costs while enhancing ‘collective problem solving’ (OECD 2005, Acharya et al. 2006, Knack and Rahman 2007, Woode et al. 2021).

The majority of evidence suggests that DAH improves health (Bendavid 2014, Yogo and Mallaye 2015) and does so through complex pathways. Evidence from reviewed studies suggests that DAH is utilized to enhance the capacity of health systems through purchasing of medical consumables and equipment, funding healthcare personnel, and building resilient health system infrastructure, thereby expanding access to healthcare (Akachi and Atum 2011, Bendavid et al. 2012, Cohen et al. 2013, Winkleman and Adams 2017). This, alongside other mechanisms such as effective aid coordination, governance, and other socio-political factors, has the potential to improve a country’s health profile.

Aid fungibility is prevalent in LMICs and a source of concern for some donors (Van de Sijpe 2013, Rana and Koch 2020). However, the few studies that have examined this in the context of the impact of DAH on health have found health outcomes to be insensitive to both full and partial fungibility (Pettersson 2007, Wagstaff 2011). The practice may therefore not be as harmful as portrayed, particularly when the objective of reallocating health aid is to optimize the welfare of the population. In this case, fungibility may be interpreted as a positive externality, especially if the additional benefits in the non-targeted sectors or programmes are greater because of the substitutability of DAH. From a DAH programming perspective, fungibility can also dictate the modalities of channelling aid to recipient countries, contingent on donors’ aims and interests. This may include earmarking aid (vertical delivery) or channelling it through public sector systems and institutions (horizontal delivery). Although the evidence is inconclusive, channelling resources through public systems appears to have some stronger effects in terms of strengthening health systems (Kostova et al. 2021, Patenaude 2021), compared to using off-budget channels such as NGOs.

Although DAH yields some health-enhancing effects, these findings must be viewed within the current context of the global health aid landscape. The 2025 funding cuts by the Trump administration starkly portray a grim picture. Stover et al. (2025) warn that these cuts could reverse hard-worn progress towards global health targets, particularly for HIV, TB, and MNCH programmes. Similarly, Cavalcanti et al. (2025) use model-based projections to estimate the impacts of eliminating US government funding. Their analyses predict 15.2 million additional AIDS deaths, 2.2 million TB deaths, 7.9 million child deaths, and 12–16 million unsafe abortions. Together, these shocks reveal three key insights: first, DAH plays a pivotal role in sustaining global health gains; second, LMICs remain vulnerable to sudden funding shifts; third, countries must strengthen domestic health financing to reduce dependence on external aid.

### Sub-group heterogeneity of effects

Health aid also significantly reduced under-five mortality among poorer households (Bendavid 2014). Looking at within-country variations, Jakubowski et al. (2017) concluded that higher levels of maternal education led to a considerable decline in under-five mortality, irrespective of the setting. This was echoed by Fichera et al. (2021), who showed that households with a better education profile and in higher wealth quintiles demonstrated greater reductions in child mortality in Zimbabwe. Within-area differences were also evident in Malawi, where deprived areas were more likely to receive aid, leading to reductions in Malaria prevalence (Marty et al. 2017). Other factors, such as fertility rates, the underlying burden of diseases, and the pattern of social expenditure, are shown to moderate the impact of DAH (Petersson 2007, Wilson 2011, Gyimah-Brempong 2015). In summary, health aid may have a protective effect on mortality and morbidity in LMICs, though its effects vary depending on governance structures, socio-economic profiles, and health aid delivery. Better targeting of DAH could be most in need.

### Strengths and limitations

This synthesis reaffirms the impact of health aid on health outcomes and suggests pathways that may maximize the effect of DAH. In general, systematic reviews are designed to filter evidence by comparing one intervention with another. However, the macro-nature of aid initiatives and disbursements implies that truly genuine comparators are challenging to identify. Hence, many of the reviewed studies relied on ‘hypothetical’ comparators, by understanding what would have happened if DAH had not been provided. While such methodologies are widely used in empirical policy evaluations (Angrist and Pischke 2009, Jones et al. 2013), they still pose methodological challenges that can distort the interpretation of findings.

Systematic reviews are also typically supported by well-validated tools that help to assess the quality of studies. This helps to achieve rigour in rating the quality of evidence and increasing its utility, especially for clinical interventions. However, in policy evaluation studies relying on observational data, the rigour of critical appraisal varies depending on the nature of the effect under consideration. As a result, we adapted and tailored tools for vital appraisal to reflect the nature of included studies. Further validation exercises are needed to assess the applicability of the tool in similar situations. The pathways through which aid improves health outcomes should be interpreted with caution and only within the scope of the included studies. Excluded studies may have had additional information on this point, though it is not captured here. Non-peer-reviewed publications, primarily from multilateral and bilateral agencies, occasionally publish reports on how the aid they provide may improve health outcomes or health systems. However, the descriptive nature of most available analyses was outside the scope of the eligibility criteria.

Despite the overall positive association between DAH and improved health outcomes, several limitations within the evidence base warrant caution. First, our review is dominated by MNCH outcomes, likely reflecting historical global health priorities, such as the Sustainable Development Goals and the Millennium Development Goals. This focus may bias the synthesis, as DAH for MNCH is often delivered alongside technical assistance and integrated programmatic support that can amplify its effectiveness. However, it is important to note that MNCH indicators are widely regarded as sensitive markers of population health and system performance. Broader outcomes, such as life expectancy and general health, are less directly linked to targeted programmes and are influenced by multiple determinants beyond DAH. These system-wide indicators may better capture the overall effect of DAH.

This review also focused on quantitative studies to enable a structured synthesis of evidence on the measurable impact of DAH, which is why qualitative studies were excluded. While this approach enhanced comparability, it may have underrepresented countries with weaker health reporting systems, where qualitative evidence is more prevalent. Thus, the absence of qualitative studies in our review may bias the findings towards better-reported health systems, warranting caution in interpreting them. Moreover, qualitative studies often provide valuable insights into the contextual factors that shape aid effectiveness, including government ownership (Oliveira Cruz and McPake 2011), financing mechanisms such as execution of SWAps, and adherence to the principles of the Paris Declaration (Biesma et al. 2009, Mwisongo et al. 2016, Nabyonga-Orem et al. 2016). While these factors can influence the success of health aid, they are beyond the scope of quantitative outcome measures. By focusing on quantifiable impacts, our review may therefore overlook nuances such as stakeholder perspectives, including more localized stories of positive or negative effects of aid, implementation challenges, and policy dynamics that qualitative studies could, in principle, shed some light on.

Quantitative studies also measure effects across a population or sub-population, which may not reflect more localized experiences of positive/negative impacts of aid funding. Thus, we make a deliberate trade-off, gaining consistency in outcomes on the one hand, while losing some depth in understanding contextual and process-related factors on the other. Future mixed-methods reviews that integrate quantitative outcomes and qualitative perspectives are needed to capture the full complexity of DAH implementation and its effects on health systems.

Methodological issues, particularly endogeneity and omitted-variable bias, persist even in studies that employ advanced econometric techniques. Reliance on self-reported data for health outcomes introduces measurement error, while discrepancies in how aid is reported complicate cross-study comparisons. Moreover, the predominance of short-term analyses may understate the full impact of aid, given the lagged effects of health interventions. Finally, publication bias, which favours studies reporting positive findings, could skew the evidence base. Addressing these challenges in future research will be critical for refining our understanding of how and under what conditions DAH is most effective.

### Future areas for research

Recent global commitments to decolonize aid and promote locally led development (GPEDC 2022, USAID 2022) highlight a critical gap in new research on the effectiveness of health aid. Whether health aid is delivered directly to communities, through local Non-Governmental organisation or Civil Society Organisation (NGOs/CSOs), or via recipient governments, assessing its impact will be both challenging and essential. Decolonizing aid can enhance its effectiveness by aligning interventions with local priorities and fostering community ownership. Importantly, it also represents a moral imperative, affirming the value of equitable partnerships. However, lessons from the Paris Declaration on Aid Effectiveness caution against neglecting the generation of evidence. Donor disengagement from the Paris Principles was partly driven by insufficient proof of their impact. This underscores the need for rigorous data collection and analysis of health aid locally led development principles.

Further, the Global Partnership for Effective Development Cooperation renewed emphasis on aid effectiveness principles provides an opportunity to examine their relevance in contemporary health aid delivery. While emerging evidence (e.g. Woode et al. 2021) suggests that adherence to these principles can improve outcomes, further research is needed to disentangle which elements are most critical for achieving sustainable health gains. This knowledge is vital for guiding donors and recipients towards more impactful and equitable health aid strategies.

The co-financing strategies implemented by the Global Fund and Global Alliance for Vaccine (GAVI) appear to stimulate domestic financing for health and hold the promise of progressively catalysing it (Global Fund 2020, GAVI 2023). By 2021, these policies are estimated to have secured $40 billion in domestic funding, aiming to transition health financing away from foreign sources gradually. However, the structure of the Global Fund Financing at the country level, through project implementation units, likely shares the inherent drawbacks of vertical funding mechanisms, such as those found in PEPFAR (Lee and Izama 2015). While the benefits seem substantial, further empirical research is required to understand their overall net effect(s). Specifically, it remains unclear whether they have improved (or not) health beyond the diseases of focus by strengthening health systems and crowding in new domestic health expenditure, or whether they have had negative externalities in some settings by displacing funding from other health-improving programmes.

The burden of disease and scope of health financing in LMICs suggest that the proliferation of global health donors and implementing agencies cannot be entirely avoided. To support effective DAH programming, further analyses could consider reviewing the impact of aid fragmentation on health financing and health outcomes. Among other issues, this could clarify the scope of transaction costs and identify strategies to streamline donor coordination and enhance efficiency in global health programming.

Most studies in the review also estimated the impact of DAH on child health outcomes, including infant and child mortality. Although these metrics represent common population health indicators, there are other relevant health dimensions as well. Health is a complex, multi-dimensional concept, and in some cases, DAH is earmarked for specific purposes. Further analyses should therefore consider unpacking the impact of disease-specific aid on the health outcomes it is specifically associated with. For example, Patenaude (2021) examined the effect of malaria-specific health aid delivered through on- and off-budget mechanisms. The study found slight declines in malaria incidence through the on-budget channel, while off-budget aid showed no significant gains. It also suggested that on-budget financing has a more substantial effect on health system performance. These findings offer relevant policy insights for decision-makers regarding the need to contextualize the impact of aid and its delivery channels. While the latter may also be an interesting finding, further analyses should explore the interplay between aid and public financial management systems, health financing and health outcomes.

We found no studies investigating the impact of health aid on Neglected Tropical Diseases (NTDs), a group of 20 debilitating conditions prevalent in tropical LMICs (WHO 2023). However, NTDs affect approximately one-sixth of the global population, causing substantial health, social and economic consequences, and accounting for 26 million DALYs in 2015 (Mitra and Mawson 2017, GBD 2020). Systematic tracking and documentation of NTD funding, as well as analysing the effects of related investments, could be vital in understanding programmatic gaps and impacts of ongoing efforts to eradicate these conditions.

## Conclusion

With the varied range of empirical studies included in this review, applying an array of statistical and econometric methods, some clarity has emerged: aid demonstrates some health-enhancing effects. However, contextual factors—from a country’s capacity to absorb available resources to the effectiveness of its political or governance system—mediate the impact of DAH. Important additional work may thus be needed to identify other tangible pathways that can maximize the effects of aid. As the evidence suggests, the flow of DAH has stabilized, though it remains sensitive to shocks and is limited in its predictability. Taken together, these factors can influence the effective planning and delivery of health services at the country level. As a result, LMICs need to develop deliberate policies to diversify and strengthen domestic healthcare financing strategies, with full consideration of equity, effectiveness, and efficiency.

## Supplementary Material

czaf102_Supplementary_Data

## Data Availability

No new data were generated or analysed in support of this research.

## Reflexivity statement

This review was led by a PhD scholar, supported by the Commonwealth Scholarship Commission, which funds scholars from developing countries. The lead author brings lived experience and professional engagement with LMIC health systems, which informed a critical, context-sensitive approach to synthesizing evidence on DAH.

The broader research team includes both senior and early career researchers with interdisciplinary expertise in global health policy, health economics, and international development. Our team reflects a balance in seniority and perspectives, with members based in both high-income and LMIC contexts. This diversity enriched our analysis, particularly in addressing issues related to donor influence and the heterogeneity of DAH outcomes.

While we aimed for inclusivity and reflexivity in our approach, we acknowledge that our team does not represent all regions or identities affected by development assistance, let alone gender balance. We remain committed to collaborative and equitable scholarship that centres the priorities and perspectives of those in aid-recipient settings.
