# Supplementary material for: The impact of official development assistance for health on health outcomes: a rapid systematic review
Source: Health Policy Plan. 2025 Dec 3;41(3):442–59. doi: 10.1093/heapol/czaf102 (PMC12972673; doi:10.1093/heapol/czaf102)
Supplement: czaf102_Supplementary_Data [file czaf102_supplementary_data.doc]

- 1. Appendix 1: Strategy Search strategy for EMBASE

| **#** | **Searches** | **Results** |
| --- | --- | --- |
| 1 | (official development assistance or official development assistance for health or ODA).tw. | 1096 |
| 2 | (development assistance for health or development assistance or external aid or foreign aid or donor resources).tw. | 1050 |
| 3 | (global health initiative* or global health financ* or donor assistance or donor programs).tw. | 516 |
| 4 | (aid adj3 (disbursement* or commitment* or flow* or international or development or project* or program*)).tw. | 9679 |
| 5 | (mortalit* or morbidit* or death* or fatalit* or prevalence or inciden*).tw. | 3229400 |
| 6 | 1 or 2 or 3 or 4 | 12022 |
| 7 | 5 and 6 | 1595 |
| 8 | limit 7 to yr="1990 - 2022" | 14 |

**Note: the search strategy was translated to run in different in different databases to yield the total number of studies**

Search strategy for web of science

("official development assistance" OR "official development assistance for health" OR "ODA" OR "development assistance for health" OR "development assistance" OR "external aid" OR "foreign aid" OR "donor resources" OR "global health initiative*" OR "global health financ*" OR "donor assistance" OR "donor programs") AND ("aid disbursement*" OR "aid commitment*" OR "aid flow*" OR "international aid" OR "development aid" OR "project aid*" OR "donor program*") AND ("mortalit*" OR "morbidit*" OR "death*" OR "fatalit*")

- 1. Econlit

| # | Searches | Results |
| --- | --- | --- |
| 1 | (official development assistance or official development assistance for health or ODA).tw. | 1096 |
| 2 | (development assistance for health or development assistance or external aid or foreign aid or donor resources).tw. | 1050 |
| 3 | (global health initiative* or global health financ* or donor assistance or donor programs).tw. | 516 |
| 4 | (aid adj3 (disbursement* or commitment* or flow* or international or development or project* or program*)).tw. | 9679 |
| 5 | (mortalit* or morbidit* or death* or fatalit* or prevalence or inciden*).tw. | 3229400 |
| 6 | 1 or 2 or 3 or 4 | 12022 |
| 7 | 5 and 6 | 1595 |
| 8 | limit 7 to yr="1990 - 2022" | 14 |

- 1. Medline

| # | Searches | Results |
| --- | --- | --- |
| 1 | (official development assistance or official development assistance for health or ODA).tw. | 1096 |
| 2 | (development assistance for health or development assistance or external aid or foreign aid or donor resources).tw. | 1050 |
| 3 | (global health initiative* or global health financ* or donor assistance or donor programs).tw. | 516 |
| 4 | (aid adj3 (disbursement* or commitment* or flow* or international or development or project* or program*)).tw. | 9679 |
| 5 | (mortalit* or morbidit* or death* or fatalit* or prevalence or inciden*).tw. | 3229400 |
| 6 | 1 or 2 or 3 or 4 | 12022 |
| 7 | 5 and 6 | 1595 |
| 8 | limit 7 to yr="1990 - 2022" | 1405 |

**Appendix 2: Data Extraction tool**

| **Author(s)** | **Year** | **Period of Analysis** | **Country** | **Country Income Status** | **Estimation Method(s)** | **No. methods applied in the paper** | **Method Typology** | **Unit of Analysis** | **Number of Units** | **No. Observations (sample size)** | **Disease Program** | **Reported Outcome** | **Intermediate Outcome(s)** | **Effect** | **Magnitude and direction of impact** | **Mechanism/Pathways** | **Controls/**  **Mediators** | **Heterogeneity of impact (within and between countries)** | **Summary of Key Finding(s)** |  |
| --- | --- | --- | --- | --- | --- | --- | --- | --- | --- | --- | --- | --- | --- | --- | --- | --- | --- | --- | --- | --- |
| **Author 1** |  |  |  |  |  |  |  |  |  |  |  |  |  |  |  |  |  |  |  |  |
| **Author 2** |  |  |  |  |  |  |  |  |  |  |  |  |  |  |  |  |  |  |  |  |
| **.** |  |  |  |  |  |  |  |  |  |  |  |  |  |  |  |  |  |  |  |  |
| **.** |  |  |  |  |  |  |  |  |  |  |  |  |  |  |  |  |  |  |  |  |
| **.** |  |  |  |  |  |  |  |  |  |  |  |  |  |  |  |  |  |  |  |  |
| **.** |  |  |  |  |  |  |  |  |  |  |  |  |  |  |  |  |  |  |  |  |
| **.** |  |  |  |  |  |  |  |  |  |  |  |  |  |  |  |  |  |  |  |  |
| **Author 60** |  |  |  |  |  |  |  |  |  |  |  |  |  |  |  |  |  |  |  |  |
| **Author 61** |  |  |  |  |  |  |  |  |  |  |  |  |  |  |  |  |  |  |  |  |

Appendix 3. Adapted RoB Assessment Tool

|  | **D1. General Questions** | **QuEENS** | **Domain Rating** |
| --- | --- | --- | --- |
| Q1, Q4 | Have different methods been compared within the study? |  |  |
| Q2 | Have the results of the study been compared to others in the literature? |  |
| Q3: | Is there a discussion of what treatment effect is identified and of the assumptions needed? |  |
| Q4 | Are the model chosen consistent with the outcome variable if using a parametric method? |  |
| Q5 | Were any checks conducted on the model specification? |  |
|  |  |  |  |
|  | **D.2 Methods assuming selection on observables** |  |  |
| Q6 | Is the assumption of selection on observables assessed? |  |  |
| Q7 | What checks were conducted to assess overlap? |  |
| Q8 | Has balancing of the covariates been checked after matching and propensity score methods? |  |
|  |  |  |  |
|  | **D.3 Methods Using Propensity Scores** |  |  |
| Q9 | Is the propensity score function sufficiently  flexible? |  |  |
| Q10 | Are potential IVs excluded from the set of conditioning variables? |  |
|  |  |  |  |
|  | **D.4 Matching method** |  |  |
| Q11 | Data quality: Are there data quality issues? |  |  |
| Q12 | For Nearest Neighbour: Has bias adjustment  been conducted if more than one variable was  included when matching on covariates? |  |
| Q13 | Is the choice of replacement (with/without) reasonable? |  |
| Q14 | Is the choice of the number of matches/callipers  matching/radius matching reasonable? |  |
|  |  |  |  |
|  | **D.5 IV Methods/GMM/ARDM** |  |  |
| Q15 | Is the instrument well justified? (i.e. eligibility in programme participation (reason), natural experiment, theoretically sensible, fitted propensity scores) |  |  |
| Q16 | Is the sample size large? |  |
| Q17 | If more than one IV, is the test of overidentifying restrictions reported? |  |
| Q18 | Is a weak instrument(s) test reported? |  |
|  |  |  |
|  | **D.6 Difference in Difference/Synthetic contol** |  |  |
| Q19 | Does the intervention generate exogenous variation? (not applicable if natural experiment) |  |  |
| Q20 | Is the assumption of common trends across groups reasonable? |  |
| Q21 | Is it reasonable to assume that there is no  selection of unobserved temporary individual/unit specific shocks? |  |
| Q22 | Is the assumption of no systematic composition changes within each group reasonable? (applicable with repeated cross-sections, not with  longitudinal data) |  |
|  |  |  |  |
|  | **D.7 Regression (Discontinuity)** |  |  |
| Q23 | Is the sample size large? |  |  |
| Q24 | Is the assumption (for RD) that individuals(units) are not able to affect the instrument to change the likelihood of participation reasonable? |  |
| Q25 | Are control variables clearly discussed? |  |
| Q26 | Is the appropriateness of the model thoroughly stated and discussed? |  |
|  |  |  |
|  | **D.8 Estimation of Outcome** |  |  |
| Q27 | Did the authors use appropriate method(s) that controlled for all important confounding domains and for time-varying confounding? |  |  |
| Q28 | We’re confounding domains validly and reliably measured by variables available in this study? |  |
| Q29 | Were intervention(treatment) group(s) or exposure clearly defined atleast from the inception? |  |
| Q30 | Were units(participants) excluded due to missing data on variables needed for the analysis? |  |
| Q31 | Is there evidence that results were robust to the presence of missing data? |  |
|  | **OVERALL QuEENS Rating** |  |  |

Appendix 4: Summary of main analytical methods applied in the analysis.

| **Method** | **Approach** | **Assumptions and validity checks** |
| --- | --- | --- |
| **Instrumental variable** | Strategy seeks to find a variable (an ‘instrument’) which is correlated with the treatment but only correlated with the outcome through its effect on the treatment. Variation in the instrument is then exploited to identify the causal effect.  Choice of an instrument is fundamental as its correlation with the treatment provides some exogenous variation to approximate randomisation | Zero-first-stage test to assess the relevance of the instrument(s) and partial F- statistics >10 implies the relevancy assumption is satisfied.  Strong correlation between the instrument and endogenous variables usually suggests the validity of the instruments, La et al (2021) |
| **Generalised Method of Moment** | Uses lagged value(s) of a given outcome variable as an instrument(s). It is an appropriate procedure if the underlying distribution of a given sample is not known and uses specific sample moments to estimate the outcomes.  Estimates derived via GMM may not be sufficient statistics given the failure to account for all information in the sample and thus inefficient in small samples | Relies on population moment conditions and uses overidentifying restriction tests to assess appropriateness of the model. The strength of GMM lies in that it leverages the dynamic nature of panel data to uncover relationships which may easily be obscured when traditional panel methods are used. |
| **Synthetic Control Method** | Used in comparative case studies and infers the impact of a program by comparing the evolution of a target outcome for a given unit affected by given policy intervention to the evolution of the same outcome in a synthetic control group.  Sometimes viewed as generalisation of DiD though unlike the former assumes that unobserved confounders change over time by re‐weighting the control group to match pre‐intervention characteristics to the treated group.  Creates synthetic control group by iterative permutations and weighting of control units to create a potential control akin to pre-intervention characteristics of the treated unit(s) | Iterative exclusion of potential control units to determine those that best approximate pre-intervention characteristics for the treated group.  Useful for health policy evaluations when the validity of the parallel trends assumption is questionable |
| **Difference in Difference** | Retrieves causal estimates based on potential outcomes framework and compares changes over time in the means of the treatment and control groups and therefore allows for both group-specific and time-specific effects.  Uses longitudinal data for the same individuals or repeated cross-sections drawn from the same population, before and after the treatment. | Parallel/common trend between the control and treatment group  Allows for selection on unobservables, it assuming no selection on the unobserved time varying individual specific shocks.  Assessing underlying assumptions such as parallel trends and absence of unobserved temporary individual specific shocks is sometimes problematic. |
| **Panel data methods** | Pooling data with a significant number of cross-sections and time series over time.  Uses individuals as their own control since everyone is observed at different time periods. | Identification of treatment effect assumes that individual unobserved heterogeneity is time invariant.  Fixed effects model or a first difference model may be applied if individual unobserved effects are assumed to be correlated with covariates in the model |

*Baltagi, 2005 ; Wooldridge, 2013 ; Faria et l, 2015 ; Abadie, 2021 ; Kreif et al, 2016

Appendix 5: Methods of selection on observables and unobservable

Empirically, several strategies are undertaken to account for endogeneity and avoid spurious causal conclusions. Broadly, these can be classified into two groups—methods of selection on observables and unobservable. The former rests in the assumption that the process of assigning units into treatment (exposed) and control (non-exposed) groups is based on observable factors that are independent of the outcome of interest and can be managed by including several control variables for those observable factors in the estimation process (Heckman and Robb 1985). In practice however, it may be difficult to account for all implicit bias. The selection on unobservable is premised on the confoundedness assumptions (Ashenfelter & Card 1985; Jones & Rice, 2011). Specifically, it considers lack of correlation between the process of assigning units to treatment and any unobservable factors. It also introduces fixed effects in the model to account for time-variant factors that may affect health outcomes (Heckman 2008; Jones & Rice, 2011; Abadie et al., 2010).

## Common controls for observable differences

Control variables are an important feature of empirical research as they help to determine the extent by which the focal covariates behave as hypothesized, i.e., strengthen internal validity of the study. They may also be classified as “good controls”, in which case they allow researchers to isolate the treatment effect by ensuring that other factors to do not influence the subsequent effect. On the other hand, “bad controls” may lead to spurious or erroneous results. While DAH was the main policy variable of interest in this study, the choice of controls varied significantly and seemed to be informed by the research question and literature. Broadly, control covariates for the included studies may be grouped as: ‘structural’—GDP, education, population growth, fertility rate, GDP per capita, etc; Political and institutional’—control of corruption, freedom index, government effectiveness and governance (Bechani & Swish 2019; Gyimah-Brempong, 2015; Negeri & Halemariam 2016); ‘health system-related’—HRH, health spending, out-of-pocket expenditure, immunisation and Insect Treated Bed Nets (ITN) coverage, urbanisation, sanitation, safe drinking water (Chauvet et al., 2012; De la Cruz,2018; Hein et al 2020) ; ‘program’—donor coordination and harmonisation, project-based or centralised aid, including modes of aid delivery Fichera et al, 2021; Han et al 2015); ‘social spending’—government health expenditures, general government expenditure on health (Oryema et al, 2017; Yogo & Mallaye, 2015).

The scope of controls reflects the complex nature of the DAH-health outcomes relationship underlining that some of the factors that affect the effectiveness of DAH are beyond the health sector. Developing relevant policies to inform effective and efficient delivery of DAH thus requires a nuanced understanding of the moderating effects and subsequent determinants of health. Table 3 summarises the main covariates and their direction of effect for part of included studies (high quality studies). In the table, the upward arrow signifies positive effects, downward arrow negative effect, the (**.**) show lack of effect while the transposed arrows represent mixed findings. The table also provides a summary of the number of countries included in the sample, the overall impact of aid as *“none, positive and negative*”, risk of bias assessment and outcomes.

| Appendix 6: Full list of studies included in the review and classification of studies by according to the Paris Principles of aid effectiveness | | | | | | | | | |
| --- | --- | --- | --- | --- | --- | --- | --- | --- | --- |
| **Author(s)** | **Publication Year** | **Period of Analysis** | **Country** | **No. Units** | **Reported Outcome** | **Effect** | **RoB** | **GRADE** | **Aid effectiveness era** |
| Boone | 1996 | 1971-1990 | Cross-country | 96 | Infant mortality | None | High RoB | High certainty | Pre Paris |
| Gomanee et al | 2005 | 1980-1998 | Cross-country | 38 | Infant mortality | Positive | Mod RoB | High Certainty | Pre Paris |
| Bhaumik | 2005 | 1995-2002 | Cross-country | 36 | Infant mortality | Positive | High RoB | Mod Certainty | Pre Paris |
| Masud & Yoncheva | 2005 | 1990-2000 | Cross-country | 58 | Infant mortality | Positive | Mod RoB | High Certainty | Pre Paris |
| Petersson | 2007 | 1978-2001 | Cross-country | 48 | Under-five mortality | Positive | High RoB | Mod Certainty | Pre Paris |
| Wolf | 2007 | 1980-2002 | Cross-country | 50 | Under-five mortality | Positive | High RoB | Mod Certainty | Pre Paris |
| Wolf | 2007 | 1980-2002 | Cross-country | 50 | Infant mortality | None | High RoB | MoD Certainty | Pre Paris |
| Burnside & Dollar | 2009 | 1970-1990 | Cross-country | 56 | Infant mortality | None | Mod RoB | High Certainty | Pre Paris |
| Mishra & Newhouse | 2009 | 1975-2004 | Cross-country | 118 | Infant mortality | Positive | Low RoB | High Certainty | Pre Paris |
| Drabo and Ebeke | 2010 | 2007 | Cross-country | 56 | Prev Diarrhoea | Positive | Low RoB | Mod Certainty | Pre Paris |
| Drabo and Ebeke | 2010 | 2007 | Cross-country | 56 | Prev Fever | Positive | Low RoB | Mod Certainty | Pre Paris |
| Akachi and Atum | 2011 | 2002-2008 | Cross-country | 34 | Under-five mortality | Positive | Low RoB | Mod Certainty | Pre Paris |
| Wagstaff | 2011 | 1997-2002 | Vietnam | 61 | Under-five mortality | Positive | Low RoB | High Certainty | Pre Paris |
| Wilson | 2011 | 1975-2005 | Cross-country | 96 | Infant mortality | None | Low RoB | High Certainty | Pre Paris |
| Bendavid et al | 2012 | 2003-2008 | Cross-country household | 27 | Adult mortality | Positive | Mod RoB | High Certainty | Pre Paris |
| Afridi and Ventolou | 2013 | 1995-2006 | Cross-country | 109 | Adult mortality | Positive | Low RoB | High Certainty | Pre Paris |
| Chauvet et al | 2013 | 1992-2004 | Cross-country | 84 | Under-five mortality | Positive | Low RoB | High Certainty | Pre Paris |
| Chauvet et al | 2014 | 1992-2004 | Cross-country | 84 | Infant mortality | Positive | Low RoB | High Certainty | Pre Paris |
| Cohen et al | 2013 | 1995-2010 | Cross-country | 41 | TB incidence | Positive | High RoB | Mod Certainty | Pre Paris |
| Cohen et al | 2013 | 1995-2010 | Cross-country | 41 | TB Mortality | Positive | High RoB | Mod Certainty | Pre Paris |
| Bendavid and Batacharya | 2014 | 1970-2010 | Cross-country | 138 | Under-five mortality | Positive | High RoB | Mod Certainty | Pre/post Paris |
| Bendavid | 2014 | 1992-2012 | Cross-country household | 49 | Under-five mortality | Positive | Mod RoB | High Certainty | Pre/post Paris |
| Burfeind | 2014 | 2000-2009 | Cross-country | 47 | Under-five mortality | Positive | High RoB | Mod Certainty | Pre/post Paris |
| Han and Koeing-Archibugi | 2015 | 1990-2010 | Cross-country | 102 | Under-five mortality | Positive | Mod RoB | High Certainty | Pre/post Paris |
| Hsiao & Emdin | 2015 | 1990-2010 | Cross-country | 99 | Mortality | Positive | Mod RoB | Mod Certainty | Pre/post Paris |
| Hsiao & Emdin | 2015 | 1990-2010 | Cross-country | 94 | Mortality | Positive | Mod RoB | Mod Certainty | Pre/post Paris |
| Gyima-Brempong | 2015 | 1990-2012 | Cross-country | 48 | Maternal mortality | Positive | Low RoB | Mod Certainty | Pre/post Paris |
| Gyima-Brempong | 2015 | 1990-2012 | Cross-country | 48 | Under-five mortality | Positive | Low RoB | Mod Certainty | Pre/post Paris |
| Gyima-Brempong | 2015 | 1990-2012 | Cross-country | 48 | Cholera mortality | Positive | Low RoB | Mod Certainty | Pre/post Paris |
| Yan et al | 2015 | 1995-2010 | Cross-country | 147 | Adult mortality | Positive | High RoB | Mod Certainty | Pre/post Paris |
| Yan et al | 2015 | 1995-2010 | Cross-country | 55 | Under-five mortality | Positive | High RoB | Mod Certainty | Pre/post Paris |
| Yogo and Mallaye | 2015 | 1990-2012 | Cross-country | 33 | HIV Prevalence | Positive | Low RoB | High Certainty | Pre/post Paris |
| Yogo and Mallaye | 2015 | 1990-2012 | Cross-country | 33 | Under-five mortality | Positive | Low RoB | High Certainty | Pre/post Paris |
| Lee & Izama | 2015 | 1998-2002 | Cross-country | 37 | Neonatal mortality | Positive | Low RoB | Mod Certainty | Pre/post Paris |
| Gillader | 2016 | 1973-2005 | Cross-country | 31 | Life expectancy | Positive | Mod RoB | Mod Certainty | Pre/post Paris |
| Odokonyero et al | 2016 | 2010-2014 | Uganda | 2300 | Disease prevalence (self-reported symptoms) | Positive | Mod RoB | High Certainty | Pre/post Paris |
| Odokonyero et al | 2016 | 2010-2014 | Uganda | 2300 | Productivity burden of disease (days of lost productivity) | Positive | Mod RoB | High Certainty | Pre/post Paris |
| Negeri and Halemariam | 2016 | 1990-2010 | Cross-country | 43 | Infant mortality | Positive | Mod RoB | Mod Certainty | Pre/post Paris |
| Ziesemer | 2016 | 1960-2000 | Cross-country | 65 | Life expectancy | Positive | Low RoB | High Certainty | Pre/post Paris |
| Pallas and Ruger | 2017 | 1995-2010 | Cross-country | 139 | Infant mortality | Positive | Low RoB | High Certainty | Pre/post Paris |
| Jakubowski et al | 2017 | 1995-2014 | Cross-country household | 32 | Under-five mortality | Positive | High RoB | Low Certainty | Pre/post Paris |
| Oryema et al | 2017 | 1998-2012 | Cross-country | 40 | Infant mortality | Positive | Mod RoB | Mod Certainty | Pre/post Paris |
| Winkleman and Adams | 2017 | 2000-2015 | Cross-country | 183 | Infant mortality | Positive | High RoB | Mod Certainty | Pre/post Paris |
| Marty et al | 2017 | 2004-2010 | Malawi | 1 | Prev Malaria | Positive | Low RoB | Mod Certainty | Pre/post Paris |
| Kenya Malaria survey | 2017 | 2003-2015 | Kenya | 1 | Mortality | Positive | High RoB | Mod Certainty | Pre/post Paris |
| Gutema & Mariam | 2018 | 1978-2013 | Ethiopia | 1 | Life expectancy | Positive | High RoB | Low Certainty | Pre/post Paris |
| Kim | 2018 | 1992-2013 | Cross-country | 40 | Adult mortality | Positive | Mod RoB | Mod Certainty | Pre/post Paris |
| Kim | 2018 | 1992-2013 | Cross-country | 40 | Ped mortality | Positive | Mod RoB | Mod Certainty | Pre/post Paris |
| Kim | 2018 | 1992-2013 | Cross-country | 30 | Peds Incidence | Positive | Mod RoB | Mod Certainty | Pre/post Paris |
| Kim | 2018 | 1992-2013 | Cross-country | 30 | Women Incidence | Positive | Mod RoB | Mod Certainty | Pre/post Paris |
| Kotsadam et al | 2018 | 1990-2013 | Nigeria | 1 | Infant mortality | Positive | Mod RoB | High Certainty | Pre/post Paris |
| Norad Evaluation Report | 2018 | 2013-2017 | India | 1 | Neonatal fever | Positive | Mod RoB | Low Certainty | Post Paris |
| Norad Evaluation Report | 2018 | 2013-2017 | India | 1 | Neonatal mortality | Positive | Mod RoB | Low Certainty | Post Paris |
| Pickbourn & Ndikumana | 2019 | 2000-2013 | Cross-country | 47 | Under-five mortality | Positive | High RoB | Mod Certainty | Pre/post Paris |
| Aknilo and Sulola | 2019 | 200-2008 | Cross-country | 10 | infant mortality | Positive | Low RoB | Mod Certainty | Pre/post Paris |
| Aknilo and Sulola | 2019 | 2000-2009 | Cross-country | 10 | Under-five mortality | Positive | Low RoB | Mod Certainty | Pre/post Paris |
| Banchani and Swish | 2019 | 1996-2015 | Cross-country | 130 | Under-five mortality | Positive | Mod RoB | High Certainty | Pre/post Paris |
| Banhart | 2019 | 2004-2014 | Kenya | 1 | Infant mortality | Positive | High RoB | Mod Certainty | Pre/post Paris |
| Jaupart et al | 2019 | 1995-2016 | Cross-country | 84 | Under-five mortality | Positive | Low RoB | Mod Certainty | Pre/post Paris |
| Jaupart et al | 2019 | 1995-2016 | Cross-country | 84 | Infant mortality | Positive | Low RoB | Mod Certainty | Pre/post Paris |
| Rustad et al | 2019 | 2001-2016 | Cross-country household | 103,138 | Weight loss | Positive | High RoB | Moderate | Pre/post Paris |
| Herzer | 2018 | 1991-2016 | Cross-country | 108 | Infant mortality | Mixed | Mod RoB | Mod Certainty | Pre/post Paris |
| Ghana Malaria Survey | 2019 | 2003-2016 | Ghana | 1 | Mortality | Positive | Mod RoB | Low Certainty | Pre/post Paris |
| Doucouliagos, et al | 2021 | 2002-2015 | Cross-country | 96 | Infant mortality | Positive | Low RoB | High Certainty | Pre/post Paris |
| Kostova et al | 2020 | 2000-2016 | Cross-country | 116 | Aggregated CVD morbidity | Positive | High RoB | Low Certainty | Pre/post Paris |
| Kostova et al | 2020 | 2000-2016 | Cross-country | 116 | Neoplasms morbidity | Positive | High RoB | Low Certainty | Pre/post Paris |
| Kostova et al | 2020 | 2000-2016 | Cross-country | 116 | CRD morbidity | Positive | High RoB | Low Certainty | Pre/post Paris |
| Kostova et al | 2020 | 2000-2016 | Cross-country | 116 | Diabetes Mellitus 1 morbidity | Positive | High RoB | Low Certainty | Pre/post Paris |
| Kostova et al | 2021 | 2000-2017 | Cross-country | 117 | Aggregated NCD mortality | Positive | High RoB | Low Certainty | Pre/post Paris |
| Kostova et al | 2020 | 2000-2016 | Cross-country | 116 | CVD mortality | Positive | High RoB | Low Certainty | Pre/post Paris |
| Kostova et al | 2020 | 2000-2016 | Cross-country | 116 | Neoplasms morbidity | Positive | High RoB | Low Certainty | Pre/post Paris |
| Kostova et al | 2020 | 2000-2016 | Cross-country | 116 | CRD mortality | Positive | High RoB | Low Certainty | Pre/post Paris |
| Kostova et al | 2020 | 2000-2016 | Cross-country | 116 | Diabetes Mellitus 1 mortality | Positive | High RoB | Low Certainty | Pre/post Paris |
| Hein et al | 2020 | 1998-2008 | Cross-country | 27 | Adult mortality | Positive | Mod RoB | High Certainty | Pre/post Paris |
| Wayoro and Ndikumana | 2020 | 1994-2012 | Coite d' Ivoire | 1 | Infant mortality | Positive | Low RoB | High Certainty | Pre/post Paris |
| Martorano et al | 2020 | 2000-2012 | Cross-country Household | 13 | Child mortality | Positive | Low RoB | High Certainty | Pre/post Paris |
| Martorano et al | 2020 | 2000-2012 | Cross-country household | 13 | Stunting | None | Low RoB | High Certainty | Pre/post Paris |
| Cruzat et al | 2020 | 2002-2014 | Cross-country | 53 | Infant mortality | Positive | Low RoB | High Certainty | Pre/post Paris |
| Woode et al | 2021 | 1990-2016 | Cross-country | 68 | Infant mortality | Positive | Low RoB | High Certainty | Pre/post Paris |
| Abraham and Tao | 2021 | 1990-2017 | Cross-country | 130 | Undernourishment | Positive | Mod RoB | Mod Certainty | Pre/post Paris |
| Abraham and Tao | 2021 | 1990-2017 | Cross-country | 130 | Maternal mortality | Positive | Mod RoB | Mod Certainty | Pre/post Paris |
| Toseef et al | 2019 | 2001-2015 | Cross-country | 90 | Adult mortality | Positive | High RoB | Mod Certainty | Pre/post Paris |
| Patenaude | 2021 | 2004-2015 | Cross-country | 134 | Under-five mortality | Positive | Mod RoB | High Certainty | Pre/post Paris |
| Patenaude | 2021 | 2004-2015 | Cross-country | 134 | Malaria Incidence | Positive | Mod RoB | High Certainty | Pre/post Paris |
| Fichera et al | 2021 | 2005-2015 | Zimbabwe | 1 | Under-five mortality | Positive | Low RoB | High Certainty | Post Paris |
| Weiss et al | 2022 | 2000-2016 | Cross-country | 25 | Under-five mortality | Positive | Low RoB | High Certainty | Pre/post Paris |
| Wolf | 2007 | 1980-2002 | Cross-country | 50 | Infant mortality | None | High RoB | Mod Certainty | Pre/post Paris |
| Nwude et al | 2019 | 1999-2017 | Cross-country | 81 | Under-five mortality | Negative | Mod RoB | High Certainty | Pre/post Paris |
| Williamson | 2008 | 1973-2004 | Cross-country | 208 | Infant mortality | None | Low RoB | High Certainty | Pre/post Paris |
| Williamson | 2008 | 1973-2004 | Cross-country | 208 | Maternal mortality | None | Low RoB | High Certainty | Pre/post Paris |
| Kizhakethalackal | 2013 | 1974-2005 | Cross-country | 110 | Infant mortality | None | Mod RoB | Mod Certainty | Pre/post Paris |
|  |  |  |  |  |  |  |  |  |  |
| Kizhakethalackal | 2013 | 1995-2005 | Cross-country | 112 | TB incidence | None | Mod RoB | Mod Certainty | Pre/post Paris |
| Wilson | 2011 | 1975-2005 | Cross-country | 96 | infant mortality | None | Low RoB | High Certainty | Pre/post Paris |
| Cohen et al | 2013 | 1995-2010 | Cross-country | 41 | Infant Mortality | None | High RoB | Mod Certainty | Pre/post Paris |
| Cohen et al | 2013 | 1995-2010 | Cross-country | 41 | Under-five mortality | None | High RoB | Mod Certainty | Pre/post Paris |
| Hsiao & Emdin | 2015 | 1990-2010 | Cross-country | 120 | Mortality | None | Mod RoB | Mod Certainty | Pre/post Paris |
| Yan et al | 2015 | 1995-2010 | Cross-country | 147 | Under-five mortality | None | High RoB | Mod Certainty | Pre/post Paris |
| Taverdi and Rammohan | 2017 | 1995-2015 | Cross-country | 79 | Under-five mortality | None | Mod RoB | High Certainty | Pre/post Paris |
| Burguet and Soto | 2017 | 2000-2008 | Cross-country | 132 | Under-five mortality | None | Low RoB | High Certainty | Pre/post Paris |
| DelaCruz | 2018 | 1995-2013 | Cross-country | 27 | HIV Prevalence | None | Low RoB | High Certainty | Pre/post Paris |
| Banhart | 2019 | 2004-2014 | Kenya | 1 | Neonatal mortality | None | High RoB | Mod Certainty | Pre/post Paris |
| Kostova et al | 2020 | 2000-2016 | Cross-country | 116 | Diabetes Mellitus 2 morbidity | None | High RoB | Low Certainty | Pre/post Paris |
| Kostova et al | 2020 | 2000-2016 | Cross-country | 116 | Diabetes Mellitus 2 mortality | None | High RoB | Low Certainty | Pre/post Paris |
| Abraham and Tao | 2021 | 1990-2017 | Cross-country | 130 | TB incidence | None | Mod RoB | Mod Certainty | Pre/post Paris |
| Toseef et al | 2019 | 2001-2015 | Cross-country | 90 | Infant mortality | None | High RoB | Mod Certainty | Pre/post Paris |
| Patenaude | 2021 | 2004-2015 | Cross-country | 134 | HIV Incidence | None | Mod RoB | High Certainty | Pre/post Paris |
| Patenaude | 2021 | 2004-2015 | Cross-country | 134 | TB Incidence | None | Mod RoB | High Certainty | Pre/post Paris |
| Fichera et al | 2021 | 2005-2015 | Zimbabwe | 1 | Infant mortality | None | Low RoB | High Certainty | Post Paris |
| Fichera et al | 2021 | 2005-2016 | Zimbabwe | 1 | Neonatal mortality | None | Low RoB | High Certainty | Post Paris |

| Appendix 7: Summary of studies by mechanism of impact | | | | | | | |
| --- | --- | --- | --- | --- | --- | --- | --- |
| **Author(s)** | **Year** | **Period of Analysis** | **Unit of Analysis** | **Number of Units** | **Reported Outcome** | **Effect** | **Pathways** |
| **Akachi and Atum** | 2011 | 2002-2008 | Country | 34 | U5 mortality | Positive | ITN and IRS coverage |
| **Yogo and Mallaye** | 2015 | 1990-2012 | Country | 33 | HIV Prevalence | Negative None | Women education completion rate and public health spending |
| **Odokonyero et al** | 2016 | 2010-2014 | Household | 2,300 | Productivity burden of disease | Positive | Proximity to aid initiatives |
| **Jakubowski et al** | 2017 | 1995-2014 | Country and household | 32 | U5MR | Positive | Coverage of key malaria interventions - ITN, IRS and ACTs |
| **Marty et al** | 2017 | 2004-2010 | Household | 1 | Malaria prevalence | Positive | Proximity to aid initiatives |
| **Kenya Malaria indicator survey** | 2017 | 2003-2015 | Household | 1 | Mortality | Positive | Coverage of key malaria interventions - ITN, IRS and ACTs |
| **Kim** | 2018 | 1992-2013 | Country | 40 | Women and pediatric HIV infections and mortality | Negative | PEPFAR funding for women and children |
| **Kotsadam et al** | 2018 | 1990-2013 | Household | 1 | Infant mortality | Positive | Geographical proximity to aid initiatives |
| **Kostova et al** | 2020 | 2000-2016 | Country | 116 | NCD outcomes | Negative | Horizontal vs vertical channels for delivering DAH |
| **Wayoro and Ndikumana** | 2020 | 1995-2014 | Household | 1 | Infant mortality | Positive | Proximity to aid initiatives |
| **Martorano et al** | 2020 | 2000-2012 | Country | 13 | U5MR& stunting | Negative | Proximity to aid initiatives |
| **Woode et al** | 2021 | 1990-2016 | Country | 68 | Infant mortality | Positive | Sector-Wide Approach (SWAP) initiative |
| **Abraham and Tao** | 2021 | 1990-2017 | Country | 130 | Stunting, TB & maternal mortality | Negative | Infrastructure and governance |
| **Fichera et al** | 2021 | 2005-2015 | Household | 1 | MNCH | Positive | Results-Based Financing |
